# From genotype to phenotype: decoding mutations in blasts by holo-tomographic flow cytometry

**DOI:** 10.1038/s41377-025-01913-y

**Published:** 2025-07-02

**Authors:** Daniele Pirone, Concetta Di Natale, Maria Di Summa, Nicola Mosca, Giusy Giugliano, Michela Schiavo, Daniele Florio, Daniela Marasco, Pier Luca Maffettone, Lisa Miccio, Pasquale Memmolo, Pietro Ferraro

**Affiliations:** 1 CNR-ISASI, Institute of Applied Sciences and Intelligent Systems “E. Caianiello”, Pozzuoli, Italy; 2 Dipartimento di Ingegneria Chimica, dei Materiali e della Produzione Industriale. Università di Napoli “Federico II”, Napoli, Italy; 3 CNR-STIIMA, Institute of Intelligent Industrial Technologies and Systems for Advanced Manufacturing, National Research Council of Italy, Bari, Italy; 4 Department of Mathematics and Physics, University of Campania “Luigi Vanvitelli”, Caserta, Italy; 5 TIGEM, Telethon Institute of Genetics and Medicine, Pozzuoli, Italy; 6 Department of Advanced Biomedical Science, University of Naples “Federico II”, Napoli, Italy; 7 Department of Pharmacy, University of Naples “Federico II”, Napoli, Italy

**Keywords:** Phase-contrast microscopy, Imaging and sensing

## Abstract

Cup-like nuclear morphological alterations in acute myeloid leukemia (AML) blasts have been widely correlated with Nucleophosmin 1 (NPM1) mutations. NPM1-mutated AML has earned recognition as a distinct entity among myeloid tumors, but the absence of a thoroughly established tool for its morphological analysis remains a notable gap. Holographic tomography (HT) can offer a label-free solution for quantitatively assessing the 3D shape of the nucleus based on the volumetric variations of its refractive indices (RIs). However, traditional HT methods analyze adherent cells in a 2D layer, leading to non-isotropic reconstructions due to missing cone artifacts. Here we show for the first time that holo-tomographic flow cytometry (HTFC) achieves quantitative specificity and precise capture of the nucleus volumetric shape in AML cells in suspension. To retrieve nucleus specificity in label-free RI tomograms of flowing AML cells, we conceive and demonstrate in a real-world clinical case a novel strategy for segmenting 3D concave nuclei. This method implies that the correlation between the “phenotype” and “genotype” of nuclei is demonstrated through HTFC by creating a challenging link not yet explored between the aberrant morphological features of AML nuclei and NPM1 mutations. We conduct an ensemble-level statistical characterization of NPM1-wild type and NPM1-mutated blasts to discern their complex morphological and biophysical variances. Our findings suggest that characterizing cup-like nuclei in NPM1-related AML cells by HTFC may enhance the diagnostic approach for these tumors. Furthermore, we integrate virtual reality to provide an immersive fruition of morphological changes in AML cells within a true 3D environment.

## Abstract (web summary)

Label-free holo-tomographic flow cytometry enables 3D analysis of nuclei in suspended cells of acute myeloid leukemia, revealing a correlation between NPM1-mutations and cup-like morphology, potentially improving diagnostics with virtual reality integration.

## Graphical abstract

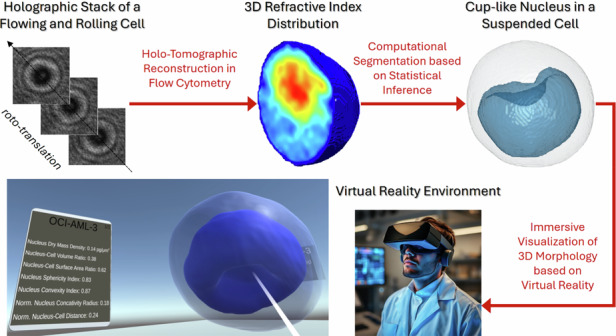

## Introduction

**Fig. 1:**
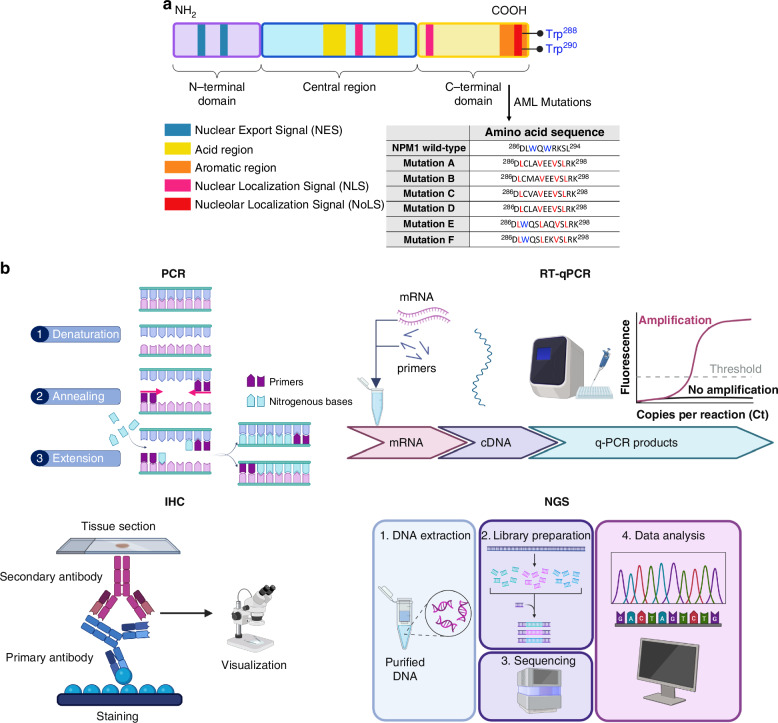
Overview of the NPM1 mutations and the conventional techniques to identify and characterize them. **a** Modular structure of NPM1-wt with NES and NOLS was evidenced. Primary sequence of wt and common AML mutations: residues in light blue constitute the NoLS, and those in red refer to the additional NES gained upon gene mutations. **b** Representative scheme of fluorescence-based tests used for the detection of NPM1-mutated AML

AML is a type of hematologic malignant tumor that grows uncontrollably from hematopoietic stem/progenitor cells. A meticulous assessment of cytogenetic mutations is required for a precise diagnosis, classification, or prognosis, as well as to determine the appropriate therapeutic approach^1^. The mutations concerning the gene that encodes for the Nucleophosmin 1 (NPM1) protein are today a reality in AML diagnosis and classification^1^. NPM1 is a multifunctional protein with a predominant nucleolar localization, endowed with a chaperone function^2–5^. This activity is regulated by the presence of nuclear export signal (NES)^6^ and NucleOlar Localization Signal NoLS stretches, where two tryptophans, Trp 288 and Trp290, constitute an uncommon NoLS^7^. NPM1 has a modular structure (Fig. 1a) and its monomers assemble as donut-shaped homo-pentamers through the N-terminal oligomerization domain (NTD). NPM1 gene mutations account for ∼30% of adult AML lesions, and the majority of mutations, ∼50 different types, concern exon 12, specifically 14 distinct mutations include the most common, types A–F, and the other 8 mutations, named J–Q^7^. These mutations are located in the C-terminal domain (CTD) of the protein and disrupt the disruption of the wild-type native three-helix bundle, and to an amyloid aggregation propensity^7–10^. All mutations are heterozygous and retain a wild type allele, they cause the loss of Trp 288 (E–F) or, more commonly, both Trp 288, 290 residues (A–D) of the NoLS, the gain of an additional NES^11^, hence they lead to an aberrant cytoplasmic localization, denoted as NPM1c+ (Fig. 1a).

In this context, distinctive molecular and clinicopathological features can be assessed, including a normal karyotype and a good response to induction chemotherapy^11^. Cytoplasmatic dislocation plays a key role in leukemogenesis, causing a dysregulation in the NPM1 interactome with both nucleic acids and several proteins^3,4,12,13^. NPM1-mutated AML has been recognized as a distinct entity in the 2017 World Health Organization (WHO) classification of myeloid tumors. However, although WHO criteria for a correct diagnosis are well established, its distinction from other AML entities is still challenged, as well as the percentage of blasts required for the diagnosis remains controversial (≥20%)^12,14^. Thus, a correct diagnosis for NPMc+ AML is extremely important and, since mutations are unable to drive clonal hematopoiesis, their presence at the remission stage indicates an active disease that could cause relapse or poor outcomes. Hence, monitoring the minimal residual disease (MRD) is central to guiding therapies at the post-remission stage, e.g., whether or not to perform allogeneic hematopoietic stem cell transplantation^14–16^.

To date, NPMc+ AML cytoplasmic detection is primarily carried out through immunohistochemistry (IHC) methods along with molecular techniques such as polymerase chain reaction (PCR) with the fragments analysis, real-time quantitative polymerase chain reaction (qRT-PCR) or next-generation sequencing (NGS), as reported in Fig. 1b^11,12,16^. In general, PCR fragment analysis methods are preferred because they can detect in a simple and rapid manner all insertions within the PCR amplicon regardless of the mutation sequence, but their sensitivity is near 5%, which is not always adequate for a differential leukemia diagnosis^16^. Qualitative assays are also applied as first-level screenings to fresh bone marrow (BM), peripheral blood (PB) leukemic cells, or the entire plasma, but they must then be integrated with other quantitative, laborious tests, resulting in wastefulness in terms of money and time^16^. Instead, molecular detection of NPM1 mutations in paraffin-embedded tissue biopsies is unreliable due to the denaturing effect of chemical agents used for tissue decalcification on nucleic acids that are essential for patients where the MRD monitoring is planned. In this case, in fact, the determination of baseline transcript levels is essential to permit comparison with post-remission samples^16^. The employment of NGS is growing for the possibility to identify NPM1 co-mutations, with a concordance > 95%, even if it is still a method with a high complexity and several technical challenges^16^. Given the increasing need to fast molecularly stratify patients (within 48–72 h to treatment initiation), novel methods are required. In this context, standard flow cytometry (FC)-based fluorescence could be a promising alternative to PCR or NGS for detecting NPM1 mutations, offering a more rapid method^16^. However, while commercially available antibodies specific to NPM1-wt exist, there are no antibodies reported for detecting mutated NPM1. The use of this antibody to observe the cytoplasmic delocalization of mutated NPM1 generates unspecific results, limiting the effectiveness of FC in diagnosing and classifying NPM1-AML^17^. Among detection techniques, IHC is almost inexpensive in the diagnostic-prognostic work-up of AML patients with normal karyotypes, and recently, IHC has been used to detect cytoplasmic NPM1 as a surrogate for NPM1 mutations involving exon 12 or other uncommon sites^18^. But, despite these positive aspects, IHC also possesses different drawbacks, e.g., the equipment is costly, stains are not standardized worldwide, and well-skilled operators are needed to avoid human errors, thus, the quantification of results is difficult. A comparison of clinical testing modalities was reported in ref. ^19^, as well as a comparison of non-sequencing techniques associated with blast morphology was reported in ref. ^20^. In this scenario, a label-free technique could bypass the laborious and time-consuming IHC staining procedures in the laboratory and could be extended to other types of biomarkers to accelerate the traditional clinical workflow.

Our hypothesis is that holographic tomography (HT) could potentially offer significant opportunities for detecting NPM1 genetic aberrations as a label-free quantitative phase imaging (QPI) method^21^. In fact, HT could provide, in principle, a quantitative analysis of the 3D morphological and biophysical alterations at the single-cell level induced by NPM1 mutations. This technique avoids the need for exogenous antibodies or other labels, because it allows 3D volumetric reconstruction of the cell’s refractive indices (RIs), which encode intracellular morphological information^22–25^. In fact, recently, 2D QPI combined with FC was able to detect AML cells from BM in a label-free way^26^. Furthermore, the conjunction between 3D HT and deep learning was proved as a rapid and cost-effective solution for the detection of NPM1-mutated AML cells^27,28^, thus furnishing new evidence that label-free tomography can overcome the 2D fluorescence-based results in this field^29^. However, it is important to note that a static HT analysis^27,28^ could lead to ambiguities regarding the true 3D shape. In fact, conventional HT systems suffer the “missing cone” problem due to their optical configuration, meaning that the axial direction is affected by a limited frequency coverage, leading to a non-isotropic 3D reconstruction^24,30^. Therefore, while static HT is a promising alternative for the blind classification of NPM1-related AML, this aspect implies significant limitations that prevent a comprehensive and accurate evaluation of the 3D morphological changes in AML due to NPM1 mutations.

To overcome the above-mentioned problem, we aim to investigate whether integrating an HT system with an FC module can provide an effective solution. Such an approach could, in principle, advance analytical technology for the detection of NPM1 mutations in leukemic blasts, thus offering a promising tool for biological studies and diagnosis of NPM1-related acute myeloid leukemia (AML). Our strategy is to investigate cells while they are flowing through a lab-on-a-chip microfluidic device by a technique named holographic tomography in flow cytometry (HTFC). HTFC has been applied in various biomedical applications^31–35^ for extracting quantitative measurements from flowing cells, including the nuclei^34^. Here we demonstrate, for the first time, that the HTFC technique can provide the identification of phenotype related to the putative genotype based on an ensemble-level statistical characterization of quantitative morphological measurements about aberrant nuclei.

In our study, OCI-AML-2 (expressing NPM1-wt) and OCI-AML-3 (expressing the type A mutation of NPMc+ and the DNMT3A R882C mutation of a DNA methyltransferase) cells are employed for intracellular analysis of NPM1-related AML^1,36^. Interestingly, in this context, different morphological features of cell nuclei are analyzed based on several works in which a “cup-like” conformation was reported for mutated blasts^1,7–9,36–39^. For example, mutated blasts were shown as medium-sized and prominent nuclear invaginations using an optical microscope by a Wright–Giemsa staining or Pappenheim staining^38,40,41^ and by transmission electron microscopy examination^38^. However, in most cells, the nucleus has a roundish morphology^42^, thus a previous version of the Computational Segmentation based on Statistical Inference (CSSI) algorithm worked properly as it was originally developed for segmenting nuclei with a convex shape within a 3D RI tomogram^34^. Nevertheless, in the case of “cup-like” nucleus alterations induced by the mutation, the previous CSSI algorithm was not suitable. Thus, here we generalize the original CSSI algorithm to allow correct 3D measurements of concave nucleus shapes. For this reason, we will refer to the original CSSI algorithm as “convex-CSSI”^34^, while we will refer to its generalization as “concave-CSSI”. By means of the concave-CSSI, a reliable characterization of the morphological and biophysical characteristics of such aberrated cup-like nuclei can be performed in 3D. Importantly, we demonstrate that the method is able to capture any small but significant differences between NPM1-wt and NPM1-mutated AML cells, which may be related to genetic aberrations.

Furthermore, in an application such as the one proposed here, where the distinctive phenotypic trait related to the genotypic alteration is the morphological change in the common convex shape of the nucleus, it becomes crucial to develop a system capable of visualizing this morphological signature. Commercial graphics software and the standard 2D screens are intrinsically limited in this sense. In fact, despite the 3D nature of the reconstructed object, they run the risk of losing morphological details that could instead be fundamental for diagnosis or biology. To overcome this problem, here we leverage virtual reality (VR) as an innovative tool that can provide a true 3D and immersive environment to three-dimensionally visualize any detail inside the biological cell^35^. VR in microscopy is an emerging and promising technology offering significant advantages in fields such as biology, medicine, and materials science^43–45^. Indeed, VR offers a unique perception of 3D data by providing a more intuitive understanding of the complex morphologies. VR visualization could improve, for example, the perception and thus the accuracy of diagnoses, thanks to a clearer view of the intracellular structures and the specific abnormal morphological features. We show that the fascinating features of VR match well with the 3D reconstructions of suspended cells furnished by the HTFC technique and with the concave-CSSI algorithm, able to segment the cup-like shapes of nuclei within the NPM1-mutated AML cells. In this immersive experience, the expert (pathologist, biologist, etc.) can easily move around the cell and, at the same time, he can suddenly dive inside the cell by virtually overcoming the barrier of cell membrane, thus observing the nuclear alterations from all the possible point of views and all the possible scales with a real-time and user-friendly modality. The reported results lay the foundations for the development of a label-free tool such as HTFC for statistical studies on the NPM1-related AML disease due to the possibility of performing blood sample analyses based on the collection of 3D and quantitative cells’ datasets and their intracellular CSSI analysis and immersive VR visualization.

## Results

### CSSI algorithm for the segmentation of concave nuclei in 3D RI tomograms of label-free suspended cells

Despite label-free microscopy such as HTFC has allowed overcoming several drawbacks of well-established marker-based microscopy due to the employment of exogenous agents, it is still limited by the lack of intracellular specificity, meaning that, within a 3D RI tomogram, there is no correlation between a delimited volume and a specific organelle^25,46^. Indeed, the standard segmentation algorithms developed for image processing are usually not suitable for retrieving the intracellular specificity in the realm of label-free QPI and HT, as different organelles often share highly overlapping distributions of their RI values^25^. For this reason, the intracellular segmentation of organelles is a challenging task in these fields. To fill the specificity gap with marker-based microscopy, in recent years, several strategies have been investigated in label-free microscopy specifically for these applications^46^. In particular, the virtual staining paradigm, firstly introduced in 2D QPI^47–49^, has also been transferred to 3D static HT^50^. In virtual staining, a deep neural network is trained by means of a dataset of co-registered 3D RI tomograms and fluorescent tomograms, to perform the organelle segmentation inside the label-free RI tomograms during the inference stage. However, finding an intracellular organelle in HTFC is much more challenging than static HT because the whole cell thickness must be scanned. At the same time, the virtual staining solution cannot be followed because of the difficulties in collecting a co-registered RI-fluorescent dataset of 3D tomograms. For this reason, recently we have implemented a statistical segmentation algorithm for HTFC (i.e., the CSSI algorithm), which allows clustering all the voxels of a certain organelle by repeating statistical hypothesis tests with respect to a reference voxel group. We demonstrated the CSSI method first for the segmentation of 3D convex shapes such as the nucleus in cancer cells^34^ and the reshaped vacuole in yeast cells^35^.

Herein, the 3D RI tomograms of 63 OCI-AML-2 cells and 63 OCI-AML-3 cells were reconstructed by using the experimental HTFC system and the corresponding numerical processing described in the “Materials and methods” section. In particular, the OCI-AML-3 cells were exploited to propose a novel algorithm for the segmentation of a concave nucleus, based on the CSSI strategy.

**Fig. 2:**
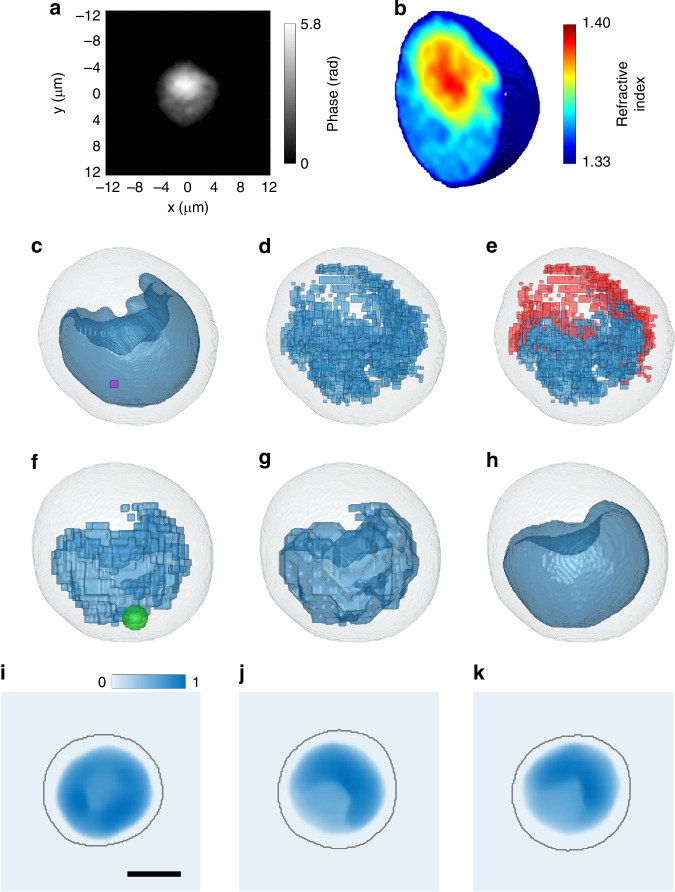
CSSI segmentation of a concave nucleus in the 3D RI tomogram of an OCI-AML-3 cell recorded by HTFC (Supplementary Movie S1 and Supplementary Movie S2). **a** QPM belonging to the overall stack of the flowing/rolling cell used to reconstruct its 3D RI tomogram. **b** Central slice of the 3D RI tomogram. **c** Nucleus initial guess (blue) obtained by segmenting the lowest values in (b) by an RI thresholding, with the reference cube highlighted in violet. **d** Intermediate nucleus set (blue). **e** Filtered intermediate nucleus set (blue) obtained by removing the outlier sub-cubes (red) from the intermediate nucleus set in (**d**). **f** Down-sampled intermediate nucleus set (blue), with highlighted in green the connection sphere used for the local linking of sub-cubes. **g** 3D nucleus polygonal (blue). **h** Final nucleus segmentation (blue), in which the concavity is indicated by the arrow. In (c-h), the cell shell is represented in gray. **i**–**k** 2D reprojections of the 3D binary nucleus segmented in (**h**) at three different orientations, normalized to their maxima. The gray line is the contour of the cell shell. Scale bar is 5 µm

The CSSI algorithm is based on a statistical hypothesis test, which is repeated several times to identify the statistical similarities between all the groups of RI voxels inside the cell (namely, test sets) and a group of voxels supposed to belong to the searched organelle (namely, reference set). The test sets having the highest statistical similarities with respect to the reference set are finally clustered to segment the searched organelle. In most cell lines with a convex nucleus, the best reference set for the nucleus segmentation is the group of central voxels inside the cell volume^34^. However, this property could be lost when the nucleus has a concave shape, because concavity could occur right at the center of the cell volume. For this reason, a different initialization of the reference set is requested for the concave-CSSI algorithm. By observing the 2D quantitative phase map (QPM) of an OCI-AML-3 cell displayed in Fig. 2a and by considering the cup-like shape of the nucleus, it can be inferred that the concave nucleus is the region with the lowest phase values inside the cell. In fact, as a first approximation, a QPM can be considered as the integral of the 3D RI distribution along the optical axis, thus encoding within a 2D image the information about the RI values of the cell coupled to its morphology. By combining multiple QPMs of the same cell acquired at different viewing angles, HT allows decoupling of this integral product, thus obtaining the 3D spatial distribution of the cell’s RIs^23^. The HTFC tomogram corresponding to the QPM in Fig. 2a (i.e., the first image of the QPM stack containing the flowing/rolling cell) is shown in Fig. 2b, where the quasi-isotropic 3D RI reconstruction can be observed due to the lack of severe missing cone problems that instead usually affect the 3D static HT^23,24,27,28,30^. To further underline this, several RI slices of the same OCI-AML-3 cell taken along different directions (i.e., *x*, *y*, and *z* axes) are displayed in the Supplementary Fig. S2, while the RI slices of an OCI-AML-2 cell are displayed in the Supplementary Fig. S3. Indeed, by comparing the 3D HTFC images in Fig. 2b, Supplementary Fig. S2, and Supplementary Fig. S3 with the 3D static HT images reported in Refs. ^27,28^ cells have similar RI values, the nucleus has lower RI values with respect to the cytoplasm, and a nucleolus with higher RI values with respect to the surrounding cytoplasm can be recognized. However, the HTFC reconstructions are quasi-isotropic in all the spatial directions, unlike the static HT cells^30^. This is confirmed by the 3D Fourier analysis reported in Supplementary Fig. S4. Indeed, as shown in Supplementary Fig. S4a, b, the HTFC system has a quasi-spherical optical transfer function (OTF), with an isotropic resolution in the *y*–*z* plane and with missing frequencies at the two opposite poles along the sole rotation *x*-axis^30^. The frequency coverage of the HTFC system is quantified in the table reported in Supplementary Fig. S4c^30^. Indeed, the cutoff frequencies are 3.12 µm^−1^ along both the flow *y*-axis and the optical *z*-axis, and 2.44 µm^−1^ along the rotation *x*-axis. Instead, as a comparison, the static HT system based on illumination scanning has a better resolution along the *y*-axis (4.88 µm^−1^), the same resolution along the *x*-axis (2.44 µm^−1^), and a worse resolution along the optical *z*-axis (1.95 µm^−1^) because of the severe missing cone issues. Thus, anisotropy in resolution is observed in the static HT system, where the ratio of minimum to maximum resolution is 0.40. The HTFC system, instead, demonstrates quasi-isotropic imaging with a resolution ratio of 0.78, indicating a substantial improvement in resolution uniformity. Furthermore, the overall OTF volume of the HTFC system (111.50 µm^−3^) is higher than the static HT system (83.37 µm^−3^) by 33.74%. Therefore, in the static HT case, there is a much higher probability of losing the cup-like shape of the nucleus as it could be randomly located in the directions affected by the missing cone artifacts. To further visualize the quasi-isotropy of the HTFC imaging, the central slices of the 3D Fourier transform about the HTFC tomogram of the OCI-AML-3 cell in Supplementary Fig. S2 and the OCI-AML-2 cell in Supplementary Fig. S3 are shown in Supplementary Fig. S4d, e, respectively.

Even if in Fig. 2b the nucleus can be recognized at the lowest RI values, a simple RI threshold is not sufficient for obtaining a reliable nucleus segmentation, as shown in Fig. 2c. Instead, the volume segmented in Fig. 2c can be exploited as nucleus initial guess to create the reference set for the CSSI algorithm. After defining a reference set appropriate for the concave nucleus segmentation, the central clustering steps of the convex-CSSI algorithm^34,35^ are implemented. In this way, the intermediate nucleus set shown in Fig. 2d is obtained, which is the cluster of sub-cubes most similar to the reference set according to the iteration of a statistical hypothesis test. Of course, the main difference between the convex-CSSI and the concave-CSSI lies in the operations for closing the selected sub-cubes to obtain the segmented nucleus. In fact, the convex-CSSI algorithm would simply connect all the pairs of selected sub-cubes, thus creating a convex 3D polygon in which the informative concavities of the nucleus are definitively lost. Instead, in the concave-CSSI algorithm, we created an ad hoc closing pipeline based on the idea that, to preserve concavities, the 3D polygonal mesh must be created locally in order to follow the real shape of the nucleus, i.e., by considering from time to time only adjacent sub-cubes instead of considering all sub-cubes together. In particular, outlier sub-cubes are deleted from the intermediate nucleus set by means of a filtering operation, thus obtaining a filtered intermediate nucleus set (see blue cubes in Fig. 2e). Then, all the possible pairs of sub-cubes in the down-sampled intermediate nucleus are locally linked to each other within a suitable volume of interest (VOI) defined by a specific criterion, thus obtaining a 3D nucleus polygonal with preserved concavities (see Fig. 2f, g). Finally, the segmented nucleus is obtained after performing a morphological closing to smooth the corners of the 3D nucleus polygonal and fill its holes (see Fig. 2h). The detailed description of the concave-CSSI algorithm is reported in the Supplementary Section S1 with a particular focus on the closing pipeline, while the intermediate tomograms generated during the several steps of the concave-CSSI algorithm (Fig. 2c–h) can be also observed from multiple viewing directions in the Supplementary Movie S1.

**Fig. 3:**
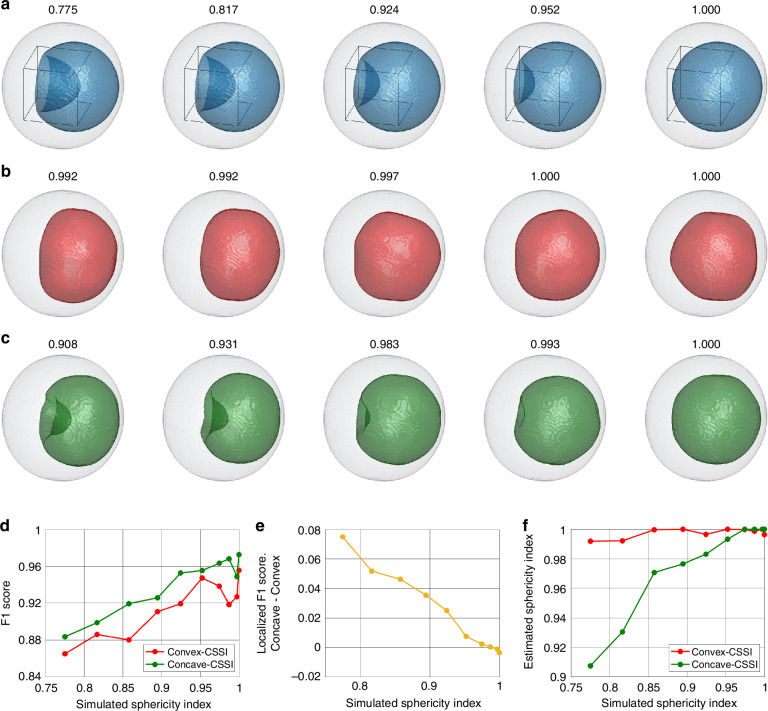
Numerical validation of the concave-CSSI algorithm. **a** Isolevels representation of some simulated nuclei (blue) within the cell shell (gray). **b** Isolevels representation of some nuclei segmented by the convex-CSSI algorithm (red) within the cell shell (gray). **c** Isolevels representation of some nuclei segmented by the concave-CSSI algorithm (green) within the cell shell (gray). In **a**–**c**, the nucleus sphericity indexes are reported at the top. **d** Comparison between the F1 scores about the nucleus segmentations obtained at different simulated sphericity indexes by the convex-CSSI and the concave-CSSI algorithms. **e** Difference between the localized F1 scores about the nucleus segmentations obtained at different sphericity indexes by the concave-CSSI and the convex-CSSI algorithms. The VOI is highlighted in (**a**) by the black box. **f** Comparison between the sphericity indexes estimated from the nucleus segmentations obtained at different simulated sphericity indexes by the convex-CSSI and the concave-CSSI algorithms

So far, no volumetric imaging of rolling cells in FC conditions has been established in the state of the art, thus, co-registration between HTFC and any reference technique cannot be performed. This means that there is no way to perform a direct comparison on exactly the same blasts with respect to gold standard techniques. Starting from this limitation, a cross-validation can be performed by an indirect comparison with well-addressed literature reporting a Wright–Giemsa staining or Pappenheim staining^27,28,38,40,41,51^ and a transmission electron microscopy examination^38^, which confirms the reliability of our results. Alternatively, to validate the proposed concave-CSSI algorithm, we performed a numerical simulation, as described in detail in the Supplementary Section S2. Indeed, by using the realistic RI distribution of the experimental cell in Fig. 2, we created a 3D numerical cell phantom made of the cytoplasm and a nucleus. In particular, starting from a perfect spherical shape, we simulated a nucleus with different concavity levels, as shown by some examples in Fig. 3a. To measure the nucleus concavity level, here we employed the sphericity index (see the section “Materials and methods”), which is 1 for a perfect spherical shape, otherwise it is lower than 1. Therefore, in Fig. 3a, for each simulated nucleus, we also report the corresponding sphericity index, which increases moving from left to right. Then, we gave the 3D RI distribution of the simulated cell to both the convex-CSSI algorithm and the concave-CSSI algorithm. As expected, while the convex-CSSI algorithm never detected nucleus concavities (see Fig. 3b), the concave-CSSI algorithm was instead able to reproduce the several levels of nucleus concavities (see Fig. 3c). To quantify this, we also computed some performance metrics. For the general assessment of the nucleus segmentation, we computed the F1 score, defined as the harmonic mean between precision (Prec) and recall (Rec)^52^, i.e.,

1 $$F1=2\frac{{Prec}\times {Rec}}{{Prec}+{Rec}}=\frac{2{TP}}{2{TP}+{FP}+{FN}}$$

where *Prec* is the probability that a pixel predicted as nucleus actually belongs to the nucleus, *Rec* is the probability of correctly segmenting a nucleus pixel, true positives (TP) is the number of nucleus pixels correctly assigned as nucleus pixels, false positives (FP) is the number of non-nucleus pixels wrongly assigned as nucleus pixels, and false negatives (FN) is the number of nucleus pixels wrongly assigned as non-nucleus pixels. As shown in Fig. 3d, for each simulated sphericity index of the nucleus, the concave-CSSI algorithm outperforms the convex-CSSI algorithm in terms of F1 score, which is on average 0.931 in the former case and 0.908 in the latter case. Instead, for a more specific assessment of the concavity segmentation, we computed a localized F1 score within the VOI containing the concave region (see the box in Fig. 3a). As reported in Fig. 3e, the discrepancy between the concave-CSSI and the convex-CSSI segmentation further becomes broader in favor of the former when the localized F1 score is calculated. Moreover, as can be expected, this discrepancy gradually decreases with the simulated sphericity index of the nucleus, until it becomes approximately zero in the case of a perfect spherical (i.e., convex) shape. However, the advantages of using the concave-CSSI algorithm are even more evident if the sphericity index estimated from the segmented nucleus is compared with the simulated one. In fact, as shown in Fig. 3f, the estimated sphericity index remains almost constant and close to 1 in the convex-CSSI. Instead, the concave-CSSI algorithm returns an increasing trend of nucleus sphericity index similar to the simulated one, which means that it can also detect and differentiate the different levels of concavities. It is also important to note that, by comparing the simulated nuclei in Fig. 3a with the segmented ones in Fig. 3c, the concave-CSSI algorithm provides a less pronounced nucleus concavity than the simulated one, as also underlined by the lower values of the estimated sphericity indexes in Fig. 3f. However, it can be expected that, as the resolution of the system will increase, the estimated concavities will align increasingly more with the real ones. Indeed, due to its working principle, the concave-CSSI algorithm can follow a concave trajectory all the better the higher the resolution with which the HTFC system has reconstructed it. In summary, the concave-CSSI algorithm generalizes the convex-CSSI algorithm because it can work for any organelle’s shape (both concave and convex). Indeed, the concave-CSSI algorithm clearly outperforms the convex-CSSI algorithm when a concave shape must be segmented, while the performance of these two algorithms becomes very similar the closer the object’s shape gets to a convex one.

As a further validation of the concave-CSSI algorithm, we also considered two well-established nucleus segmentation methods, i.e., the Cellpose algorithm^53^ and the Region Growing algorithm^54^, the latter belonging to the 3D point cloud segmentation methods^55^. The segmentation performances of these two algorithms are reported in Supplementary Fig. S5 in the case of the 3D numerical cell phantom. In particular, the Cellpose algorithm used in the nucleus modality always segmented a convex nucleus shape (see Supplementary Fig. S5b) regardless of the simulated nucleus concavity (see Supplementary Fig. S5a). Instead, the Region Growing method shares the same conceptual idea behind the concave-CSSI method. In fact, its segmentation process iteratively compares RI voxel values by starting from a seed voxel, which is here selected as the voxel corresponding to the RI median value of the nucleus initial guess (see step 1 in Supplementary Section S2). For this reason, as shown in Supplementary Fig. S5c, the Region Growing algorithm is able to segment the nucleus concavity much better than the convex-CSSI algorithm and the Cellpose method, and also better than the concave-CSSI performance, as quantified by the localized F1 score and the estimated sphericity index displayed in Fig. S5e, f, respectively. However, when considering the overall nucleus segmentation, the concave-CSSI algorithm outperforms the Region Growing algorithm, as quantified by the F1 score in Supplementary Fig. S5d (average F1 score of 0.931 vs 0.862, respectively). More generally, the Region Growing algorithm has several limitations, as its performance heavily relies on the initial seed selection and on the thresholding settings. Above all, it is very sensitive to noise and provides poor output when working with low-contrast images. For these reasons, as shown in Supplementary Fig. S6, when implemented over the experimental OCI-AML-3 cell of Fig. 2 reconstructed by HTFC, thanks to its robust statistical working principle combined to a geometric management of concave boundaries, only the proposed concave-CSSI algorithm is able to segment a reliable concave nucleus shape in very good agreement with the expected one in this cell type (see Supplementary Fig. S6d), while the Region Growing algorithm leads to a completely unrealistic nucleus segmentation (see Supplementary Fig. S6b).

### 3D morphological/biophysical characterization of concave nuclei in suspended AML cells

Combining the proposed concave-CSSI algorithm with the HTFC technique is crucial to detect nucleus concavities. In fact, working in the 3D space allows overcoming the inaccuracy of 2D imaging in reproducing the actual nucleus shape. To highlight this, we reprojected along several directions the binary 3D nucleus segmented by the concave-CSSI algorithm in Fig. 2h. In particular, we summed up the binary 3D nucleus of Fig. 2h, thus obtaining the 2D reprojection displayed in Fig. 2i–k. These 2D reprojections emulate the appearance that the nucleus would have in 2D imaging. In 2D microscopy, usually only one image per cell is acquired at a random orientation. However, by observing Fig. 2i–k, it can be seen that, even if the reprojections refer to the same cell, the information about the nucleus concavity can be lost (Fig. 2i) or difficult to detect depending on the nucleus orientation (Fig. 2j, k). Hence, for each cell orientation, different information about the nucleus concavity can be grasped, which can lead to ambiguous conclusions about this property when a single image is collected. This can be better observed in Supplementary Movie S2, where hundreds of reprojections are reported, obtained by rotating the binary 3D nucleus around the three Cartesian axes.

**Fig. 4:**
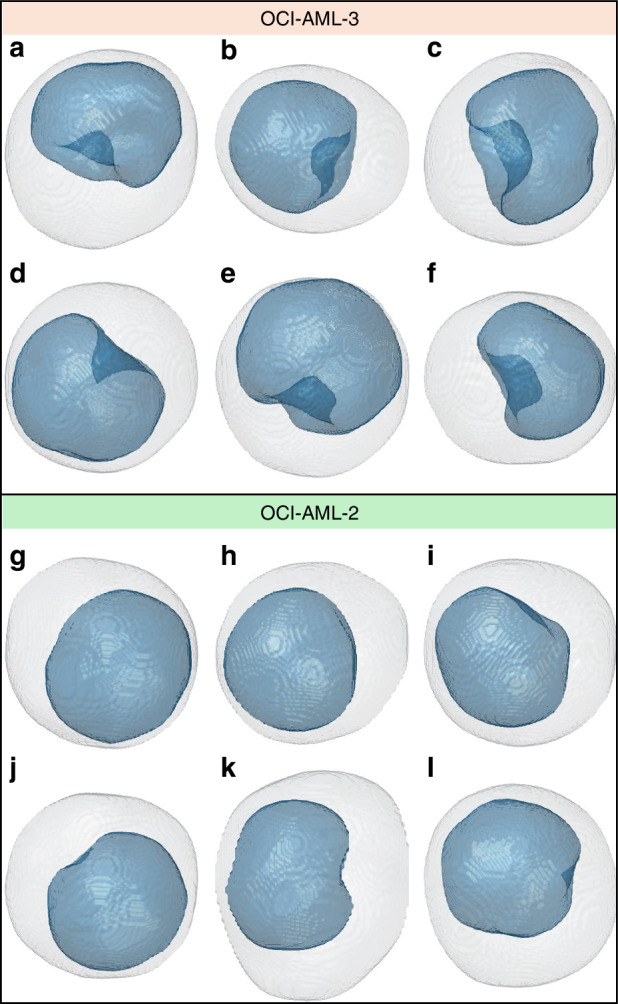
3D stain-free nucleus segmented in **a**–**f** 6 OCI-AML-3 cells (Supplementary Movie S3) and **g**–**l** 6 OCI-AML-2 cells (Supplementary Movie S4) by the concave-CSSI algorithm. The segmented nucleus is represented in blue, while the cell shell is represented in gray

Instead, the HTFC analysis allows for to elimination of this ambiguity since the same 3D image contains the overall information about the nucleus concavity along all the viewing directions. This can be seen in Fig. 4a–f, where we report the 3D nucleus segmentation of some OCI-AML-3 based on the concave-CSSI algorithm. In Fig. 4a–f, the 3D analysis provided by HTFC can be even more appreciated since it can be seen that, even if all OCI-AML-3 cells have a concave nucleus, the degree of concavity differs from cell to cell. Moreover, in addition to the intraspecies variability, we also examined the interspecies variability by segmenting the 3D stain-free nuclei in OCI-AML-2 cells, some of which are displayed in Fig. 4g–l. As a general observation, the OCI-AML-2 nuclei also exhibit a certain concavity in their 3D shape, even if it is less evident than the OCI-AML-3 cell line^50,56^. These data are in perfect agreement with those reported in the literature in which NPM1 mutations were identified in 86% of cases of AML samples with cuplike nuclei, a frequency significantly higher than the control group, in which the cup morphology was evaluated only for 19% of cases^57^. Moreover, a ROC analysis showed that a number of cup nuclei blasts ≥ 10% is highly linked to an NPM1 mutation, but the presence of 1–6% modified blasts was also found in half of the cases negative for NPM1 mutations^58^.

**Fig. 5:**
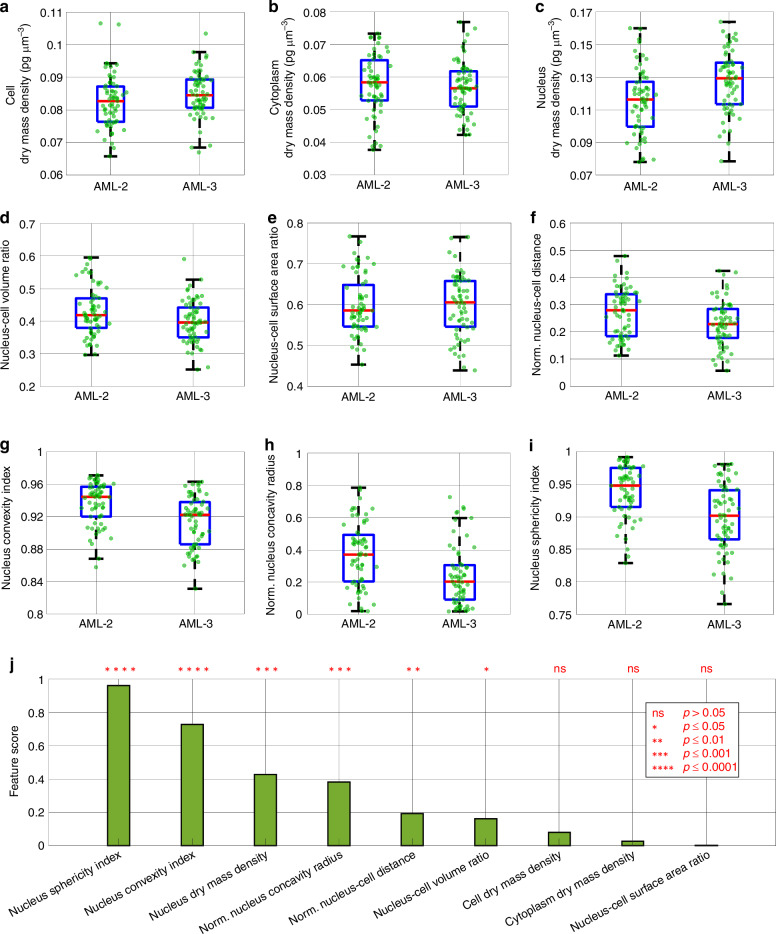
Biophysical/morphological features measured from the 3D RI tomograms of 63 OCI-AML-2 cells and 63 OCI-AML-3 cells segmented by the concave-CSSI algorithm. **a**–**i** Boxplots of the biophysical/morphological features. The central red line is the median. The bottom and top blue edges are the 25th and 75th percentiles, respectively. The black whiskers extend to the most extreme non-outlier data points. The green dots are the measured features. **j** FDR scores used to rank the features in (**a**–**i**). For each feature, the corresponding *p*-value is reported at the top

It is important to note that, unlike conventional 3D static HT in which cells are squeezed on a substrate, in 3D HTFC, the whole thickness of the cell is reconstructed since it is recorded in suspension inside a microfluidic device. For this reason, the real 3D concavity of the nucleus is reproduced as it actually is. Thus, HTFC allows performing the most reliable 3D measurements of both morphological and biophysical cell parameters. The box plots of some features measured from the 3D RI tomograms of 63 OCI-AML-2 cells and 63 OCI-AML-3 cells are reported in Fig. 5 (see the section “Materials and methods”). A typical biophysical parameter that can be obtained in HT is the dry mass, that is, the mass of a certain sample without considering its aqueous content, which can be correlated to the state of the cell and possibly to a certain disease condition^21^. By normalizing the dry mass to the sample volume, its dry mass density can be computed, which can be expressed in picograms per cubic micron. When considering the dry mass density of the whole cell, OCI-AML-3 cells have slightly higher values than OCI-AML-2 cells (see Fig. 5a). Of course, the cell dry mass density is given by the sum of two contributions, i.e., the cytoplasm and the nucleus. However, it happens that OCI-AML-3 cells have a slightly lower dry mass density with respect to OCI-AML-2 cells in terms of cytoplasm (see Fig. 5b), while their nuclei have higher values of dry mass density (see Fig. 5c). This suggests that the NPM1 mutation has induced a dry mass density increase of the nucleus differently from the cytoplasm, which density has decreased. Instead, as for the 3D morphology of the nucleus, OCI-AML-3 cells have a slightly lower nucleus–cell volume ratio with respect to OCI-AML-2 cells (see Fig. 5d). This can be explained by the more concave shape of their nuclei, which also leads to a slightly higher nucleus–cell surface area ratio (see Fig. 5e). Finally, the normalized nucleus–cell distance is the distance between the cell centroid and the nucleus centroid normalized to the nucleus equivalent radius, that is the radius of a sphere having the same volume as the analyzed nucleus. As reported in Fig. 5f, the OCI-AML-3 nuclei are closer to the cell centroid with respect to the OCI-AML-2 nuclei, which are instead more displaced towards the cell membrane.

However, to quantify the concavity degree of the 3D segmented nuclei, some ad hoc parameters have been computed. In particular, the nucleus convexity index in Fig. 5g is here defined as the volume ratio between the nucleus and its convex hull, i.e., the smallest 3D convex region containing the nucleus. The convexity index is 1 for a perfect convex object, otherwise, it is lower than 1. Instead, the normalized nucleus concavity radius in Fig. 5h is here defined as the distance between the nucleus centroid and the closest point belonging to the nucleus shell, normalized to the nucleus equivalent radius. Hence, the less pronounced the concavity, the lower the normalized nucleus concavity radius, which is 1 in the case of a perfect sphere. Finally, the nucleus sphericity index in Fig. 5i is defined as the ratio between the surface area of a sphere having the same volume as the analyzed nucleus and its actual surface area. Hence, the sphericity index is 1 for a spherical object, otherwise, it is lower than 1. As shown in Fig. 4, nuclei in OCI-AML-3 cells are more concave than the OCI-AML-2 nuclei, (see also Supplementary Movie S3 and Supplementary Movie S4, in which the same segmented cells are shown during their roto-translation inside the microfluidic channel, as occurs during the HTFC experiments). For this reason, the nucleus convexity index, the normalized nucleus concavity radius, and the nucleus sphericity are significantly lower in the OCI-AML-3 case, thus quantitatively confirming the qualitative and visual analysis carried out in Fig. 4. It is important to note that the nucleus sphericity and the normalized nucleus concavity radius are able to describe well the degree of concavity if the object is not elongated, as occurs in the AML nuclei, otherwise low values could not be related to the object’s concavity.

To rank 3D features in Fig. 5a–i according to their ability in differentiating OCI-AML-2 and OCI-AML-3 cells, we calculated Fisher’s discriminant ratio (FDR)^59^ as feature score, defined as

2 $$\begin{array}{c}{\rm{FDR}}=\frac{{\left({\mu }_{2}-{\mu }_{3}\right)}^{2}}{{\sigma }_{2}^{2}+{\sigma }_{3}^{2}}\end{array}$$

where ${\mu }_{2}$ and ${\sigma }_{2}$ are the mean value and standard deviation of the OCI-AML-2 feature, and ${\mu }_{3}$ and ${\sigma }_{3}$ are the mean value and standard deviation of the OCI-AML-3 feature. Moreover, for each feature in Fig. 5a–i, we also carried out the two-sample Student’s *t*-test^60^. In Fig. 5j, features are ranked according to their FDR scores, and the corresponding *p*-values are reported at the top. A precise conformity between the FDR values and the *p*-values exists. In particular, only in the nucleus–cell surface area ratio, cytoplasm dry mass density, and cell dry mass density, there is no statistical significance in the differences observed between the OCI-AML-2 and OCI-AML-3 populations, and these three features also have the lowest FDR scores. Instead, the most discriminating features are, in order, the nucleus sphericity index, the nucleus convexity index, the nucleus dry mass density, and the normalized nucleus concavity radius, which also show a very high statistical significance in the differences observed between the OCI-AML-2 and OCI-AML-3 populations. This confirms that the 3D concavity of the nucleus observed through HTFC and segmented by the concave-CSSI algorithm is a distinctive discriminating factor to detect OCI-AML-3 cells, as well as the nucleus dry mass density.

### Immersive visualization of cup-like nuclei through VR

We developed a VR environment to make available a tool for advanced visualization that lets the user view the various cells in a completely immersive 3D realm. The user can conduct evaluations and comparisons among actual cells in an interactive way, facilitating and improving the output of these analyses. The usefulness of the VR instrument is even more evident when the biological object has a peculiar intracellular morphological complexity that needs to be inspected and detected.

The case of cup-like nuclei in NPM1-mutated AML cells is an example of a biomedical study that could take great advantage of the potentialities of VR combined with HTFC and the concave-CSSI. To prove this, we considered the OCI-AML-3 cell in Fig. 4c as an example of an NPM1-mutated cell and the OCI-AML-2 cell in Fig. 4j as an example of an NPM1-wt cell. In particular, the HTFC tomograms segmented through the concave-CSSI algorithms were given as input to the VR tool. The VR application was developed using Unity 2021.3 (https://unity.com/products/unity-engine), and it ran on a workstation featuring an NVIDIA Quadro A4500 and an HTC VIVE Pro 2 (https://www.vive.com/us/product/vive-pro2/overview/). For testing and capturing videos/images, another workstation with an NVIDIA 3080 and an HTC VIVE Pro 2 was utilized.

**Fig. 6:**
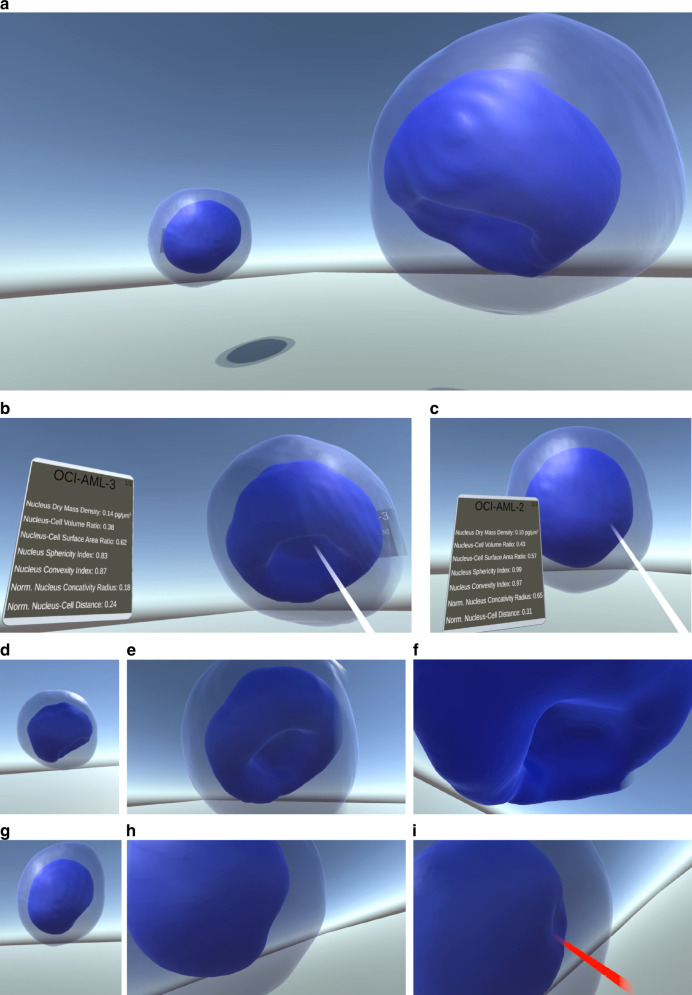
Views of the user immersed in the VR environment to inspect the AML cells reconstructed by HTFC and segmented by the concave-CSSI (Supplementary Movie S5). **a** Overall view of the OCI-AML-3 cell (on the right) and the OCI-AML-2 cell (on the left). **b**, **c** Tablet activated by the user reporting some measurements about the pointed nucleus of the OCI-AML-3 cell and the OCI-AML-2 cell, respectively. **d**–**f**, **g**–**i** The user moves toward the OCI-AML-3 cell and the OCI-AML-2 cell, respectively, to better appreciate the nucleus morphology. In **f**, the user overcomes the cell membrane, thus entering the OCI-AML-3 cell to better observe the degree of concavity of the cup-like nucleus

This secondary setup aimed to enhance immersion, featuring a room-scale play area and incorporating the VIVE Wireless Adapter (https://www.vive.com/eu/accessory/wireless-adapter-full-pack/) to free the user from any cable management. This configuration allowed users to move freely within the virtual environment and “teleport” to new locations for longer distances. For the graphical representation, tomograms were processed to achieve higher-resolution meshes. Blood cells from OCI-AML-2 and OCI-AML-3 classes were positioned approximately one meter apart in the environment. The samples were also placed at head height for optimal viewing. Considering the freedom provided by the room-scale play area, the user can crouch a bit should they need to observe the sample from below. Supplementary Movie S5 shows an example of immersive visualization experienced by a user wearing a VR headset. Some representative screenshots from Supplementary Movie S5 are instead displayed in Fig. 6. The cytoplasm is displayed with a semi-transparent material, allowing visibility through it, while the nucleus is shown in a solid blue color as the primary focus of the investigation. The starting positioning in the virtual space of the OCI-AML-3 cell (on the right) and OCI-AML-2 cell (on the left) can be seen in Fig. 6a. The user can interact with the object by means of a laser pointing, thus activating the visualization of a tablet showing the quantitative measurements about the selected object, i.e. the nucleus in this case study. In Fig. 6b, c, some of the parameters measured in Fig. 5 can be seen over the tablet for the OCI-AML-3 cell and the OCI-AML-2 cell, respectively. Moreover, unlike common visualization tools that only provide a flat view of a 3D object, VR allows users to enjoy it from different perspectives and scales by just walking around the virtual environment, thus changing the point of view in a very natural way.

For example, in Fig. 6d, g, the user is approaching the OCI-AML-3 cell and the OCI-AML-2 cell, respectively. In Fig. 6e, h, the user is right in front of the OCI-AML-3 cell and the OCI-AML-2 cell, respectively. But the most fascinating event occurs in Fig. 6f, where the user, after noticing the peculiar nuclear concavity from the outside, jumps inside the OCI-AML-3 cell by overcoming the cell membrane to arrive very close to the point of interest in order to carry out an in-depth inspection of this morphological alteration. In fact, in this way, the user can realize in real-time the actual entity of the nucleus concavity and conduct an interactive and dynamic comparison with the OCI-AML-2 cell exhibiting a much less marked concavity (Fig. 6i).

## Discussion

The hot discovery of a high correlation between NPM1 mutations and distinct nuclear structures described as “cup-like” suggested that nuclear 3D morphology analysis may be helpful in predicting the putative genotype of NPM1 blasts, thus being prognostically significant. This aspect guided us to investigate the reliability of this correlation by using a label-free methodology. To date, indeed, the diagnosis of NPM1-related AMLs is still based on different levels of screening, which involves the use of several traditional bio-molecular techniques such as PCR, q-RT-PCR, and so on. Although these methodologies are well-established at clinical level, none of them still act for the real gold standard in terms of specificity, sensitivity, and time consuming^7–9,11–16,37^. Starting from these considerations, we achieved a detailed 3D reconstruction of cell nuclei based on an HTFC system. The quantitative 3D analysis of morphological aberrant alterations in blasts induced by a genetic anomaly such as the NPM1 mutation was very valuable. In fact, we demonstrated that combining the HTFC technique with the processing based on the CSSI algorithm is a winning strategy. We showed that the approach is an effective solution capable of providing an advanced analytical tool for NPM1 mutations in leukemic blasts, possibly resulting in a new diagnostic tool for AML. Our technique surpasses static HT^27,28^ by enabling dynamic cell imaging in suspension, facilitating high-throughput analysis through FC, and ensuring accurate 3D shape reconstruction that eliminates artifacts caused by cell adhesion to flat surfaces. In fact, although the nucleus may be less sensitive than the cytoplasm to stress while on a glass slide, an adherent cell culture could promote nuclear stretching by modifying its real morphology. This aspect could be of great relevance in the context where even small nuclear morphological alterations could have a putative diagnostic power. Moreover, AML blood cells live in suspension, thus, analyzing them using a flow cytometry system such as HTFC is preferable over static adhesion-based methods since it allows for preserving their native state. It is also important to note that the fluid dynamic conditions imposed to induce the cell rotation typical of the HTFC paradigm do not alter the conformation of the cell and, consequently, the morphology of its nucleus. Furthermore, the ability of HTFC imaging to overcome the significant missing cone problems inherent in static HT^24,30^ is fundamental for the application herein demonstrated. Indeed, the shape concavity typical of NPM1-mutated AML blasts usually occurs on one side of the nucleus. In static HT, there is a high probability that this information is lost, whether the concavity is randomly located in the missing cone direction. Instead, the quasi-isotropic reconstruction provided by HTFC allows for solving this uncertainty factor.

As proof-of-concept, the OCI-AML-2 and OCI-AML-3 cells were studied as NPM1-wt and NPM1-mutated AML cell lines, respectively. As in HTFC, no chemical staining is employed to mark intracellular organelles, we exploited a computational and statistical strategy for segmenting the nucleus, namely the CSSI algorithm. Starting from the original version of the CSSI method demonstrated for 3D convex shapes^34^, we extended it for the first time to 3D concave shapes. As in principle an organelle does not have a perfect convex shape, the concave-CSSI algorithm can be considered a crucial step forward in the accurate segmentation of intracellular organelles within the HTFC reconstructions, since it can work for any organelle’s shape (both concave and convex). Here we demonstrated the concave-CSSI algorithm at the aim of detecting concavities of cup-like nuclei typical of NPM1-mutated AML^1,7–9,37–39^. By exploiting the nuclei segmentations in 3D RI reconstructions of living and suspended cells, we carried out a reliable characterization of the real 3D morphological features at the nucleus level. In this way, along with a 3D comprehensive visual analysis, we quantified the cup-like shape of NPM1-mutated AML nuclei by means of ad hoc 3D concavity features. The same features were also able to detect concavities in NPM1-wt AML nuclei, even if less marked than NPM1-mutated AML cells, as also reported in literature^51,56^. At the same time, the phase-contrast working principle at the basis of HTFC allowed recognizing differences in terms of dry mass density between NPM1-wt and NPM1-mutated AML cells, thus suggesting not only morphological changes, but also biochemical variations in their composition. In fact, in NPM1-mutated AML cells, the dry mass density increases inside the nucleus, while it decreases inside the cytoplasm. This result can be due to two different factors: (i) NPM1-AML mutations are always heterozygous, hampering the total absence of nucleolar NPM1, and (ii) the concomitant DNMT3A R882C mutation, which can further affect nuclear pathways and composition^61^. Following the successful demonstration of the concave-CSSI algorithm for concave nuclei segmentation, we will explore its extension to other 3D intracellular organelles within HTFC cell tomograms, aiming to achieve multi-specific organelle detection.

Furthermore, this study is the first to employ VR for visualizing AML blasts, thanks to the concave-CSSI nucleus segmentation in 3D HTFC reconstructions of suspended cells. This combination enables precise capture of nuclear morphologies and real-time interactive exploration of cup-like concavities in AML cells, particularly those with NPM1 mutations. In fact, we developed a VR environment to fully exploit the information contained within the 3D RI tomogram of an AML cell recorded in suspension by HTFC and segmented by the concave-CSSI algorithm^35,43–45^. VR offers to the expert (biologist, pathologist, etc.) an immersive, room-scale exploration based on the possibility to walk around and inside the AML cell in a natural and user-friendly way, thus facilitating the collection of morphological information about the nucleus concavity representing a phenotypic biomarker of the genetic NPM1 mutation. This immersive experience allows the user to go beyond the barrier of the cell membrane and observe the cup-like nucleus from all possible points of view and scales, thus overcoming limitations of conventional visualization tools, which can only flatten the 3D objects. Moreover, real-time display of quantitative data alongside visualization (e.g., via interactive virtual “tablets”) provides immediate access to critical measurements. The VR environment is particularly advantageous for studying biologically complex structures, such as the concave nuclei in AML cells, where visualizing concavity from the outside and “entering” the nucleus creates an unprecedented level of morphological understanding. For this reason, in the developed VR tool, the different degrees of concavity between an NPM1-wt and an NPM1-mutated AML cell become much more evident. Thus, the ability to “enter” the nucleus and closely analyze the concavity by a dynamic and user-guided inspection demonstrates a novel approach to correlating morphological changes with specific genetic mutations. This “genotype-phenotype” link has not been explored in such detail before and could open promising opportunities for improving clinical diagnostic workflows. In the following, some possible scenarios are discussed, which could convert VR from the visualization tool presented herein to a transformative element in diagnostic workflows, enhancing morphological analysis, fostering collaborations, and ultimately paving the way for more precise, efficient, and personalized approaches to disease diagnosis and treatment. VR could offer a streamlined way to consolidate diagnostic data, integrating imaging, genetic information, and quantitative metrics into a single interactive environment. This could significantly enhance workflow efficiency, reducing the fragmentation of medical data across different platforms and improving the speed and accuracy of clinical decision-making. For example, VR could integrate genetic data with cellular morphology, enabling a unique opportunity to correlate genotype with phenotype, thus offering a more dynamic way to analyze disease mechanisms and potentially refine classification criteria. Additionally, by enabling remote diagnostics through telemedicine, VR has the potential to expand access to advanced pathology analysis, allowing specialists to provide consultations and second opinions from anywhere in the world. Indeed, beyond individual case analysis, VR could facilitate real-time collaborative diagnostics by allowing multiple specialists to simultaneously examine and discuss the same 3D reconstructions, regardless of their physical location. This shared virtual space could enhance multidisciplinary decision-making, particularly for complex cases requiring expertise from different fields. The future of VR in diagnostics also extends to non-invasive disease monitoring and personalized treatment simulations. Instead of relying solely on physical biopsies, clinicians could explore high-resolution 3D models derived from imaging data, tracking morphological changes over time to assess disease progression or treatment response. This virtual approach could reduce the need for repeated invasive procedures while providing continuous insights into a patient’s condition. Moreover, VR could allow physicians to simulate therapeutic interventions, observing in real time how different treatments might influence nuclear morphology and overall cell behavior, thus aiding in personalized treatment selection.

In summary, this work lays the foundation for a new generation of 3D, label-free, quantitative imaging flow cytometry, addressing a notable gap in AML diagnostics. Our investigation aimed to demonstrate the proof-of-concept of a novel 3D label-free method to detect nuclei concavities representing the morphological biomarker able to distinguish OCI-AML-2 and OCI-AML-3 cells. In particular, we conducted an ensemble-level characterization by analyzing two populations of single-kind blasts and statistically pinpointing the one with specific NPM1 mutations. The attained results open the way for potential clinical validation of the technique, which will require additional studies to prove that the proposed method can phenotype single unknown cells into specific blasts with a given genotype, thus allowing single-cell differentiation on a cell-to-cell basis. Our vision for the future is to validate these observations on real pediatric and adult patient samples affected by NPM1-mutated AML. We will test our methodology to identify different genetic alterations of NPM1 by analyzing a dense network of morphological changes between nucleoli, nucleus, and cytoplasm. It is indeed widely reported that different mutated NPM1 proteins hold different cellular localizations, emptying or overcrowding a certain cell compartment^7–9,15,37^. In routine clinical practice where testing for several genetic abnormalities or rapid testing is not achievable, recognizing distinctive morphologic findings by our proposed FC technology, which is label-free, quantitative, 3D, and high-throughput, could allow the pathologist to rationalize or arrange appropriate molecular tests in a second-level screening. In addition, VR microscopy can be combined with artificial intelligence to analyze large HTFC datasets more effectively, identifying fine details that might be missed by traditional methods.

## Materials and methods

### Sample preparation

OCI-AML-2 and OCI-AML-3 cells were grown in alpha-MEM (Euroclone, Milan, Italy) supplemented with 20% fetal bovine serum, 100 U mL^−1^ penicillin, and 10 µg mL^−^^1^ streptomycin sulfate and cultured in a humidified incubator, at 37 °C, in a 5% *v*/*v* CO_2_ atmosphere. All chemical reagents used for treatments were supplied from Sigma-Aldrich (St. Louis, MO, USA) unless otherwise specified. For viability measurements ×10^3^ cells were seeded in 96-well plates and counted by Trypan Blue exclusion. All viability assays were performed in triplicate or quadruplicate and reproduced at least twice in independent experimental sessions.

### HTFC experiments

**Fig. 7:**
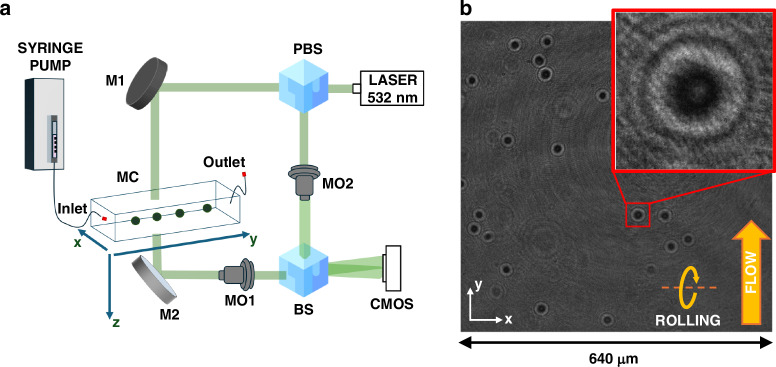
Label-free technique for recognizing NPM1 mutations in AML. **a** Sketch of the HTFC recording system. PBS polarizing beam splitter, M mirror, MO microscope objective, MC microfluidic channel, BS beam splitter, CMOS camera. **b** The 5120 × 5120 digital hologram taken from the recorded HTFC video sequence, with a highlighted in red a 384 × 384 holographic ROI. According to the reference system, cells flow along the *y*-axis and rotate around the *x*-axis

To perform HTFC experiments, a digital holography (DH) microscope in off-axis configuration has been implemented based on a Mach–Zehnder interferometer, as sketched in Fig. 7a^33^. The laser (Laser Quantum Torus 532) is used as an illumination source for emitting a light beam at 532 nm, which is split by a polarizing beam splitter (PBS) into object and reference beams. The two beams are driven across two different paths. The object beam illuminates the microfluidic channel (Microfluidic ChipShop 10000107 − *L*_*z*_ = 200 μm, *L*_*x*_ = 1000 μm, *L*_*y*_ = 58.5 mm) while cells are flowing along the *y*-axis and rotating around the *x*-axis thanks to the hydrodynamic forces of a laminar flow generated at ~50 nL s^−1^ by an automatic syringe pump (CETONI Syringe Pump neMESYS 290N). The object beam is then collected by the Microscope Objective (MO1, Zeiss Plan-Apochromat 40×, numerical aperture = 1.3, oil immersion), while the reference beam follows a free path and is collected by another microscope objective (MO2, NewPort 20×, numerical aperture = 0.40). Both collimated beams are recombined by a beam splitter cube (BS) with a non-null angle, according to the off-axis definition. The generated interference pattern propagates up to a CMOS camera (Genie Nano-CXP Cameras—5120 × 5120 pixels—4.5 μm pixel size), which records the holographic video sequence at 30 fps by covering a field of view (FoV) of 640 μm × 640 μm. The experiment consists of recording a long-time holographic video sequence of tens of minutes of the entire FoV. At the aim to retrieve one tomogram for each cell, we need a full 360° tumbling, which occurs approximately in the range of 0.5–1.0 s. However, as shown in Fig. 7b, our FoV of 640 μm × 640 μm allows for imaging tens of cells simultaneously, meaning that hundreds of cells per minute pass in parallel across the FoV.

An example of recorded 5120 × 5120 digital hologram can be seen in Fig. 7b. Starting from the full-FoV hologram, all flowing and rotating cells are detected and tracked by an automatic algorithm operating on regions of interest (ROIs) of sizes 384 × 384, as highlighted in red in Fig. 7b. Once identified the centroids of a cell, a sequence of cropped holograms measuring 1024 × 1024 pixels are used to reconstruct the corresponding QPMs^33^. These sizes allow for avoiding cutting the available information bandwidth, thus preserving the resolution. Initially, the cropped hologram is demodulated using a Fourier-domain bandpass filter by exploiting the off-axis configuration. In DH, as cells are usually recorded out of focus, their in-focus positions are numerically computed by minimizing an image contrast-based metric calculated by propagating the demodulated hologram along the optical *z*-axis using the Angular Spectrum formula. Then, the argument of the in-focus complex wavefront is extracted from which residual phase aberrations are compensated for by subtracting a reference hologram recorded without any sample in the microfluidic channel. Denoising and unwrapping algorithms are applied to obtain the QPM (see Fig. 2a). Because of the cell rotation, hundreds of QPMs are imaged for each cell at multiple viewing angles, which are estimated from the corresponding y-positions. Finally, the filtered back projection (FBP) algorithm is fed by the QPMs stack and the corresponding viewing angles to reconstruct the 3D RI tomogram^33^. In particular, we used the FBP algorithm as tomographic reconstruction algorithm since, in a technique like HTFC able to ensure the quasi-isotropic tomographic reconstruction (i.e., reduced missing cone issues), it is the best trade-off between computational burden and quality of the tomographic reconstruction with respect to other algorithms based for example on diffraction models.

The reconstruction of the 3D RI tomogram takes 10–20 min in total for each cell, considering that hundreds of QPMs must be processed. In particular, this HTFC processing time can be seen as the sum of three terms, i.e., the cell’s detection/tracking time, the QPMs reconstruction, and the tomographic reconstruction (including the viewing angles’ estimation and the FBP algorithm). Given that the cell’s detection/tracking takes less than 1 min^32^ and that the tomographic reconstruction takes a few seconds, most of the processing time is due to the QPMs reconstruction time^32,62^. However, we have already demonstrated that the QPMs reconstruction can be sped up by 45 times through deep learning^62^, which would have a great positive impact on the whole HTFC processing.

### Features calculation

The dry mass density is calculated as

3 $${\rm{Dry\; mass\; density}}=\frac{\bar{n}\left(x,y,z\right)-{n}_{0}}{\alpha }$$

where $\bar{n}\left(x,y,z\right)$ is the mean value of the RI distribution $n\left(x,y,z\right)$ of the object (e.g., the cell, the cytoplasm, or the nucleus), ${n}_{0}$ is the RI of the surrounding medium (here considered as the water’s RI, i.e. $1.334$), and $\alpha$ is the refractive increment (here assumed as 0.19 mL g^−1^ ^21^).

The nucleus–cell volume ratio is calculated as

4 $${\rm{Nucleus}-{cell\; volume\; ratio}}=\frac{{V}_{N}}{{V}_{C}}$$

where *V*_*N*_ the nucleus volume and *V*_*c*_ is the cell volume.

The nucleus–cell surface area ratio is calculated as

5 $${\rm{Nucleus}-{cell\; surface\; area\; ratio}}=\frac{{S}_{\rm{N}}}{{S}_{\rm{C}}}$$

where *S*_N_ is the nucleus surface area, and *S*_C_ is the cell surface area.

The normalized nucleus–cell distance is calculated as

6 $${\rm{Normalized\; nucleus}}-{\rm{cell\; distance}}=\frac{\sqrt{{\left({x}_{N}-{x}_{C}\right)}^{2}+{\left({y}_{N}-{y}_{C}\right)}^{2}+{\left({z}_{N}-{z}_{C}\right)}^{2}}}{R}$$

where $\left({x}_{N},{y}_{N},{z}_{N}\right)$ is the nucleus centroid, $\left({x}_{C},{y}_{C},{z}_{C}\right)$ is the cell centroid, and $R$ is the nucleus equivalent radius, defined as

7 $$R=\root{3}\of{\frac{3{V}_{N}}{4\pi }}$$

The nucleus convexity index is computed as

8 $${\rm{Nucleus\; convexity\; index}}=\frac{{V}_{\rm{N}}}{{V}_{\rm{H}}}$$

where ${V}_{H}$ is the volume of the convex hull of the nucleus, i.e., the smallest 3D convex region containing the nucleus.

The normalized nucleus concavity radius is computed as

9 $${\rm{Normalized\; nucleus\; concavity\; radius}}=\frac{\sqrt{{\left({x}_{N}-{x}_{S}\right)}^{2}+{\left({y}_{N}-{y}_{S}\right)}^{2}+{\left({z}_{N}-{z}_{S}\right)}^{2}}}{R}$$

where $\left({x}_{S},{y}_{S},{z}_{S}\right)$ is the closest point to the nucleus centroid among those belonging to the nucleus shell.

The nucleus sphericity index is computed as

10 $${\rm{Nucleus}}\; {\rm{sphericity}}\; {\rm{index}}=\frac{\root{3}\of{\pi {\left(6{V}_{N}\right)}^{2}}}{{S}_{N}}$$

## Supplementary information

Supplementary file

Supplementary Movie S1

Supplementary Movie S2

Supplementary Movie S3

Supplementary Movie S4

Supplementary Movie S5

## Data Availability

The authors declare that all data supporting the findings of this study can be found within the paper and its Supplementary Information files. Additional data supporting the findings of this study are available from the corresponding authors upon reasonable request.
