# Supplementary material for: From genotype to phenotype: decoding mutations in blasts by holo-tomographic flow cytometry
Source: Light Sci Appl. 2025 Jul 2;14:233. doi: 10.1038/s41377-025-01913-y (PMC12222964; doi:10.1038/s41377-025-01913-y)
Supplement: Supplementary file 1 — Supplementary file [file 41377_2025_1913_MOESM1_ESM.pdf]

Supplementary Information of

From Genotype to Phenotype: Decoding Mutations in Blasts by Holo-Tomographic Flow Cytometry

Daniele Pirone,[1] Concetta Di Natale,[1,2,*] Maria Di Summa,[3] Nicola Mosca,[3] Giusy Giugliano,[1,4] Michela Schiavo,[1,4,5,6] Daniele Florio,[7] Daniela Marasco,[7] Pier Luca Maffettone,[2] Lisa Miccio,[1,*] Pasquale Memmolo,[1,*] and Pietro Ferraro[1]

[1] CNR-ISASI, Institute of Applied Sciences and Intelligent Systems "E. Caianiello", Via Campi Flegrei 34, 80078 Pozzuoli, Napoli, Italy.

[2] Dipartimento di Ingegneria Chimica, dei materiali e della produzione industriale. Università di Napoli "Federico II", P.le Tecchio 80, 80125 Napoli, Italy.

[3] CNR-STIIMA, Institute of Intelligent Industrial Technologies and Systems for Advanced Manufacturing, National Research Council of Italy, Via Amendola 122/D-O, 70125 Bari, Italy.

[4] Department of Mathematics and Physics, University of Campania "Luigi Vanvitelli", Viale Abramo Lincoln 5, 81100 Caserta, Italy.

[5] TIGEM, Telethon Institute of Genetics and Medicine, Via Campi Flegrei 34, 80078 Pozzuoli, Napoli, Italy.

[6] Department of Advanced Biomedical Science, University of Naples "Federico II", Via Sergio Pansini 5, 80131 Napoli, Italy.

[7] Department of Pharmacy, University of Naples "Federico II", Via Domenico Montesano 49, 80131 Napoli, Italy.

* pasquale.memmolo@isasi.cnr.it ; lisa.miccio@isasi.cnr.it ; concetta.dinatale@unina.it

S1. Concave-CSSI algorithm

In this section we will describe the several steps of the concave-CSSI algorithm.

1. A nucleus initial guess is segmented by considering all the RI values lower than a threshold T_0 set as the q_0 quantile of the 3D RI distribution.

As the concavities of the nucleus shape must be reproduced, a better resolution is needed when clustering the several test sets, otherwise they could be lost and represented as convex curves.

2. The $L_x \times L_y \times L_z$ array containing the cell is numerically up-sampled s times [35].
3. Cell is centered in its $sL_x \times sL_y \times sL_z$ array and then divided into distinct cubes (i.e., investigated or test cubes) with an edge measuring ε pixels (step 1 in Ref. [34]).

4. A reference set is fixed as the ε-cube belonging to the nucleus initial guess having the median values closest to a random value extracted from the uniform distribution $U\{a,b\}$, where a and b are the q_a and q_b quantiles of the nucleus initial guess (see the red cube in Fig. 2c).

After defining the reference set, the core of the convex-CSSI algorithm is implemented (steps 2-5 reported in the Supplementary Information of Ref. [34]), as briefly described in the following.

5. The RI values of each investigated ε-cube are compared to those of the reference ε-cube by means of a non-parametric hypothesis statistical test, i.e. the Wilcoxon-Mann-Whitney (WMW) test [S1,S2] (steps 2-3 in Ref. [34]).
6. A preliminary partial nucleus set is created by the investigated ε-cubes having the highest p-values (steps 2-3 in Ref. [34]).
7. A filtering and refining of the preliminary partial nucleus set is performed in order to obtain a partial nucleus set made of sub-cubes with an edge measuring $\varepsilon/2$ pixels (steps 4-5 in Ref. [34]).
8. K partial nucleus sets are obtained after repeating K times steps 5-7. Indeed, every time, a random selection of the reference set is performed, thus the K partial nucleus sets are slightly different from each other.

Of course, the main difference between the convex-CSSI and the concave-CSSI lies in the operations for closing the selected sub-cubes to obtain the segmented nucleus.

9. An intermediate nucleus set is created by considering all the sub-cubes (i.e., $\varepsilon/2$-cubes) clustered at least once within the K partial nucleus sets (see Fig. 2d).
10. Outlier sub-cubes are deleted from the intermediate nucleus set by means of a filtering operation, thus obtaining a filtered intermediate nucleus set (see blue cubes in Fig. 2e). Outlier sub-cubes are defined as sub-cubes too far from the other ones (see red cubes in Fig. 2e).
    a. For each sub-cube belonging to the intermediate nucleus set, the average distance with respect to all the other sub-cubes is computed, thus creating a vector $\bar{d}_1$.
    b. All sub-cubes for which

$$\bar{d}_1 - Me(\bar{d}_1) > T_1 \quad \text{(S1)}$$

is true are removed, with

$$T_1 = -\frac{1}{\sqrt{2}erfcinv(3/2)} Me(|\bar{d}_1 - Me(\bar{d}_1)|) \approx 1.48 Me(|\bar{d}_1 - Me(\bar{d}_1)|) \quad \text{(S2)}$$

where $Me(\cdot)$ is the median value, $|\cdot|$ is the absolute value, and $erfcinv(\cdot)$ is the inverse complementary error function.

11. The filtered intermediate nucleus set is down-sampled s times to the original $L_x \times L_y \times L_z$ size, thus obtaining the down-sampled intermediate nucleus set (see Fig. 2f). The $\varepsilon/2$-cubes forming the down-sampled intermediate nucleus set are those $\varepsilon/2$-cubes in which at least one of its corresponding 8 $\varepsilon/2$-cubes belonged to the filtered intermediate nucleus set.
12. All the possible pairs of $\varepsilon/2$-cubes in the down-sampled intermediate nucleus are locally linked to each other to obtain a 3D nucleus polygonal (see Fig. 2g).
    a. For each $\varepsilon/2$-cube belonging to the down-sampled nucleus set, the q_2 quantile of its distances with respect to all the other cubes is computed, thus creating a vector $\bar{d}_2$.
    b. For each $\varepsilon/2$-cube belonging to the down-sampled nucleus set,
        i. a connection sphere having a radius equal to the average value of the vector $\bar{d}_2$ is centered in the selected cube (see green sphere in Fig. 2f);
        ii. all the possible pairs of cubes in the connection sphere are linked through a line segment.
13. The segmented nucleus is finally obtained after performing a morphological closing [S3] to smooth the corners of the 3D nucleus polygonal and fill its holes (see Fig. 2h).

The intermediate tomograms generated during the several steps of the concave-CSSI algorithm (Figs. 2c-h) can be also observed from multiple viewing directions in the Supplementary Movie S1. Parameters employed in the concave-CSSI algorithm are reported in Table S1.

S2. Simulation of the 3D numerical cell phantom

In this section we will describe the simulation of the 3D numerical cell phantom employed to validate the concave-CSSI algorithm. As shown in Fig. 3, we simulated nuclei with different concavity levels within the same cell shell. To emulate the experimental scenario, for each cell phantom we created a $201 \times 201 \times 201$ array with a pixel size of 0.125 μm.

As for the cytoplasm, we simulated an ellipsoid centered in the origin of the coordinate reference system with radii $R_{C,x} = 48$ px, $R_{C,y} = 50$ px, and $R_{C,z} = 52$ px, to emulate the real cell size (see Fig. S1a). Moreover, to simulate the cell membrane, we morphologically dilated the cytoplasm ellipsoid by 3 px (see Fig. S1a) [S3].

Instead, to simulate the concave nucleus N, we exploited the intersection between two non-concentric ellipsoidal shapes, i.e. $N = N_1 \,\&\, (1 - N_2)$, where & is the logical and. In particular, N_1 was fixed as a sphere centered in $\left(C_{N_1,x}, C_{N_1,y}, C_{N_1,z}\right) = (10,0,0)$ with a radius $R_1 = 40$ px (green sphere in Fig. S1b), while N_2 was simulated as an ellipsoid centered in $\left(C_{N_2,x}, C_{N_2,y}, C_{N_2,z}\right) = (a, 0,0)$ with radii $R_{N_2,x} = 40$ px, $R_{N_2,y} = 25$ px, and $R_{N_2,z} = 25$ px (red ellipsoid in Fig. S1b). Indeed, by intersecting N_1 and $1 - N_2$, the concave nucleus N can be obtained, as displayed in Fig. S1c.

After simulating the compartments' volumes, we assigned them realistic RI distributions inspired by the experimental OCI-AML-3 cell of Fig. 2. Indeed, from the experimental OCI-AML-3 cell of Fig. 2 we measured a RI distribution of $n_N = 1.359 \pm 0.008$ for the nucleus, $n_C = 1.363 \pm 0.014$ for the cytoplasm, and $n_M = 1.339 \pm 0.005$ for the cell membrane (obtained after a morphological erosion of the cell shell by 3 px [S3]). Then, for each compartment volume, we extracted the RI values from a Gaussian distribution having the mean and standard deviation values measured from the corresponding experimental compartments. Finally, to make the RI distributions more continuous, for each compartment volume we performed a down-sampling and an up-sampling by 4 times, followed by an average filtering of the overall cell phantom by a $5 \times 5 \times 5$ kernel. The central slice

of the resulting 3D simulated cell phantom is shown in Fig. S1d, while the corresponding RI histogram is reported in Fig. S1e.

To simulate various concavity levels of the nucleus, we considered 10 values of the a parameter (i.e., $C_{N_2,x}$), corresponding to different sphericity indexes, as reported in Table S2. If $a = 70$, there is no overlapping between N_1 and N_2, thus the nucleus N has a perfect convex (and spherical) shape.

Table S1. Parameters of the concave-CSSI algorithm. Parameters employed in the concave-CSSI algorithm to segment the stain-free concave nucleus from the 3D RI tomograms of flowing AML cells recorded by HTFC.

$L_x = L_y = L_z = 200\, pixels$	$\varepsilon = 8\, pixels$	$s = 2$	$K = 20$
$q_0 = 0.75$	$q_a = 0.25$	$q_b = 0.75$	$q_2 = 0.01$

Table S2. Parameters to simulate the nucleus concavity in the 3D numerical cell phantom. Different a values and corresponding sphericity indexes of the nucleus.

$C_{N_2,x} = a$	25	30	35	40	45	50	55	60	65	70
$sphericity\ index$	0.775	0.816	0.858	0.894	0.924	0.952	0.974	0.987	0.997	1.000

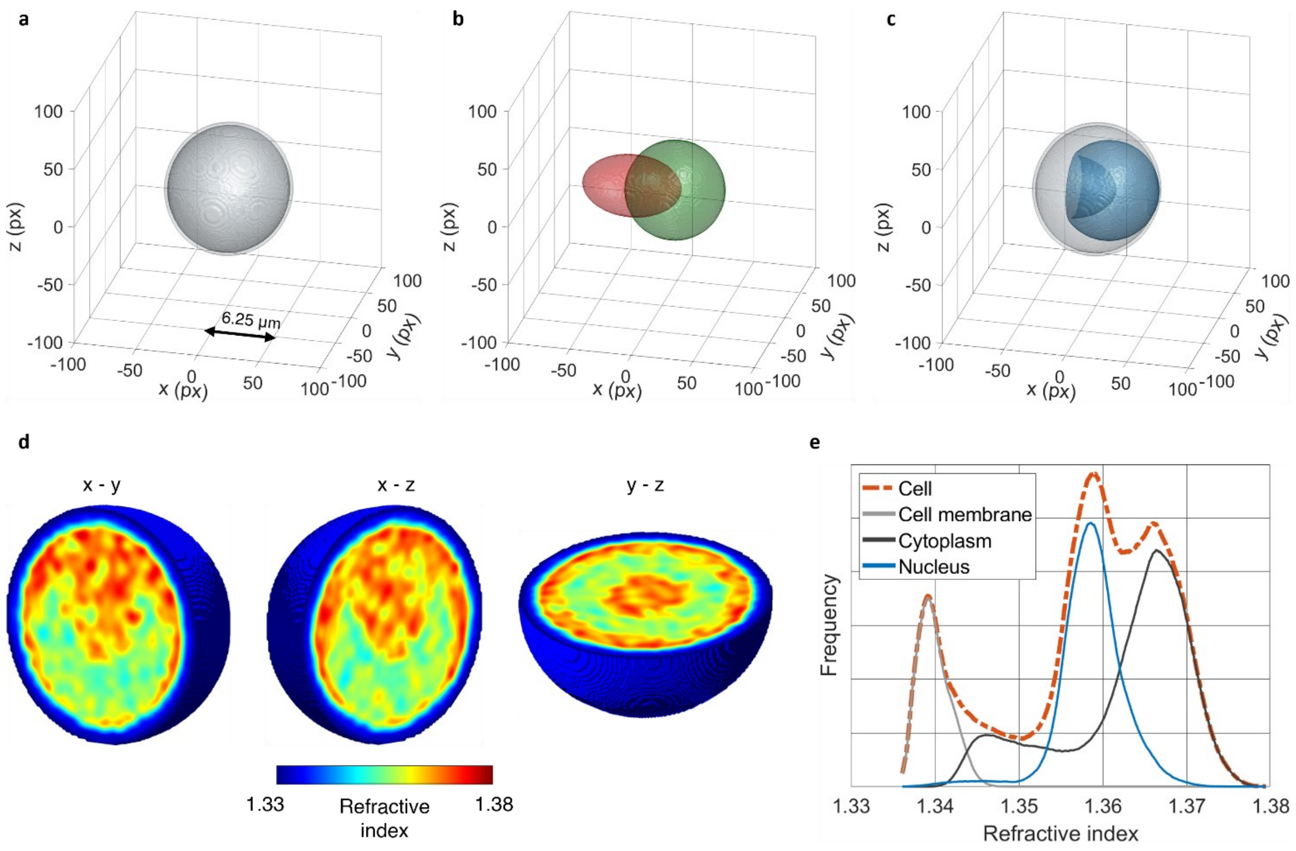

Fig. S1. 3D numerical cell phantom simulated with $a = 20$ px. **a** Shell of the cytoplasm (dark gray) and the cell membrane (light gray). **b** Shell of the N_1 volume (green) and the N_2 volume (red). **c** Simulated nucleus (blue) within the cytoplasm (dark gray) and the cell membrane (light gray). **d** Central slices of the 3D numerical cell phantom in (c). **e** Histogram of the RI distribution of the 3D numerical cell phantom in (c) and its compartments.

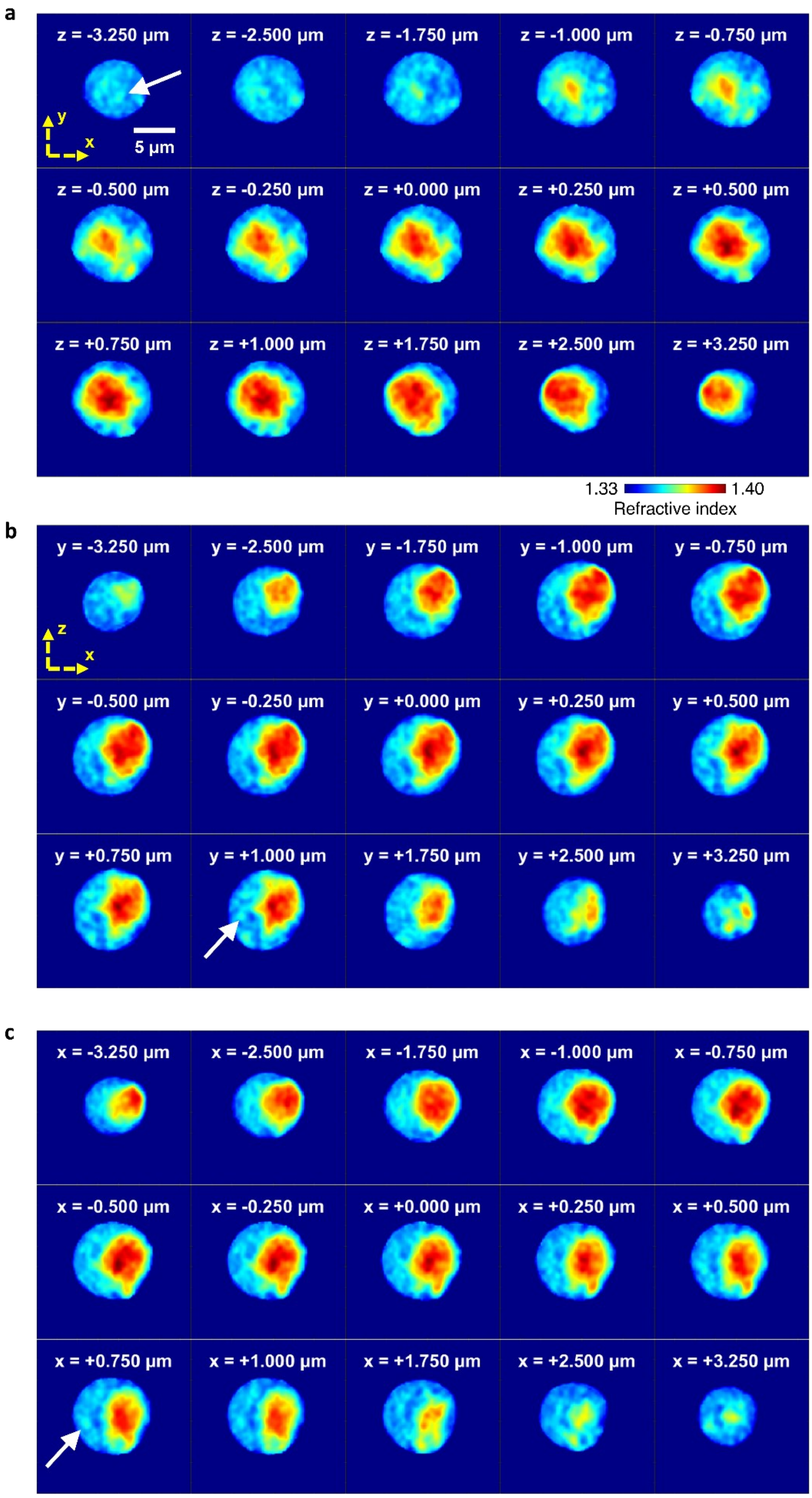

Fig. S2. Several slices of the 3D RI tomogram of an OCI-AML-3 cell reconstructed by HTFC taken along different directions. HTFC allows a 3D quasi-isotropic reconstruction not affected by the missing cone problems. a Section xy. **b** Section yz. **c** Section yz. The white arrow indicates a nucleolus inside a volume with low RIs corresponding to the nucleus.

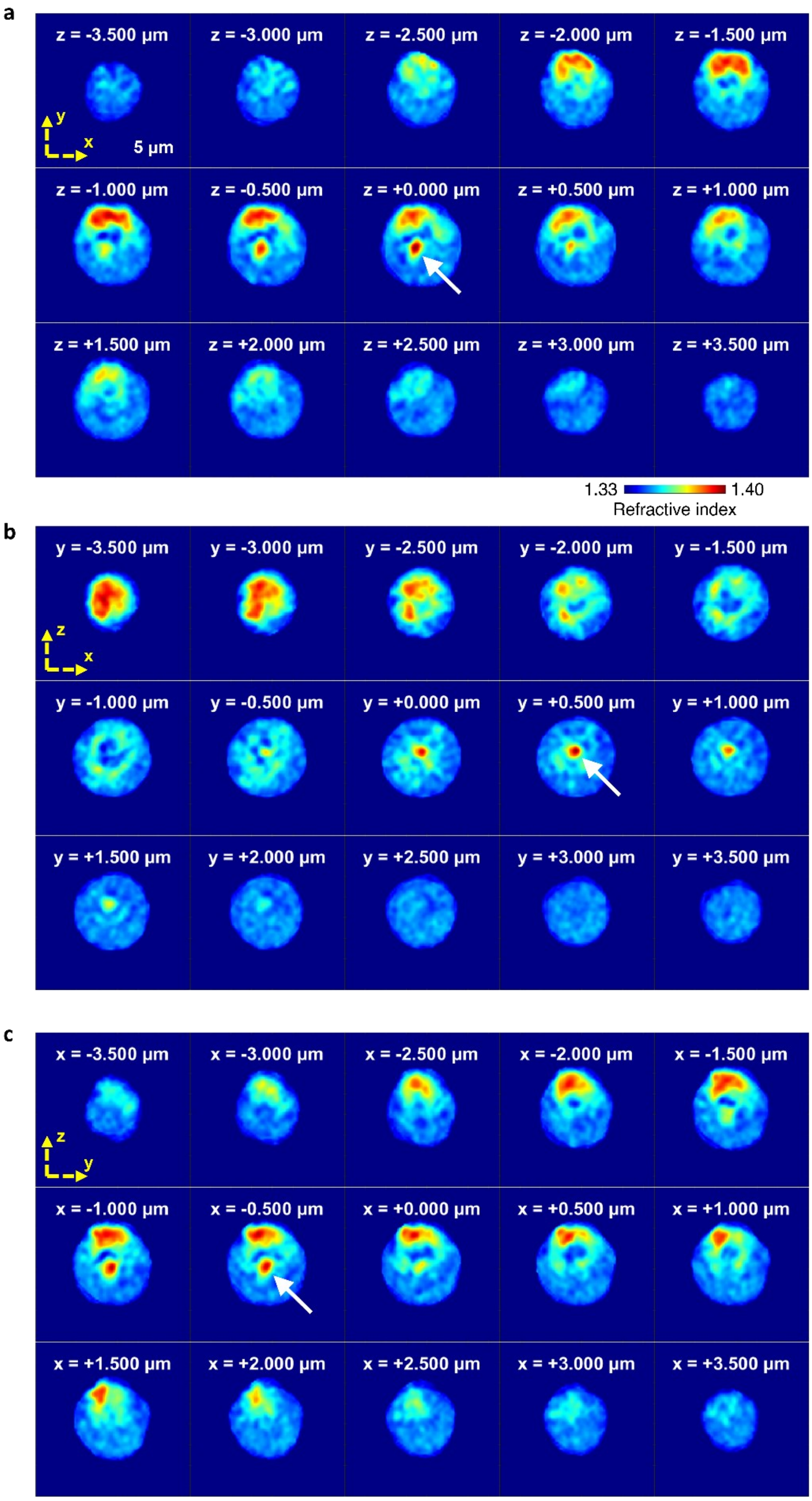

Fig. S3. Several slices of the 3D RI tomogram of an OCI-AML-2 cell reconstructed by HTFC taken along different directions. HTFC allows a 3D quasi-isotropic reconstruction not affected by the missing cone problems. **a** Section xy. **b** Section yz. **c** Section yz. The white arrow indicates a nucleolus inside a volume with low RIs corresponding to the nucleus.

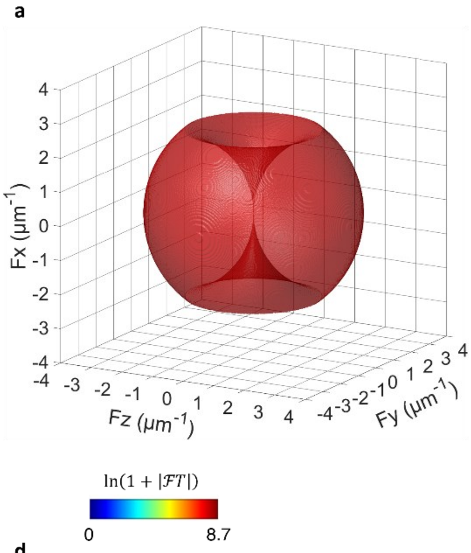

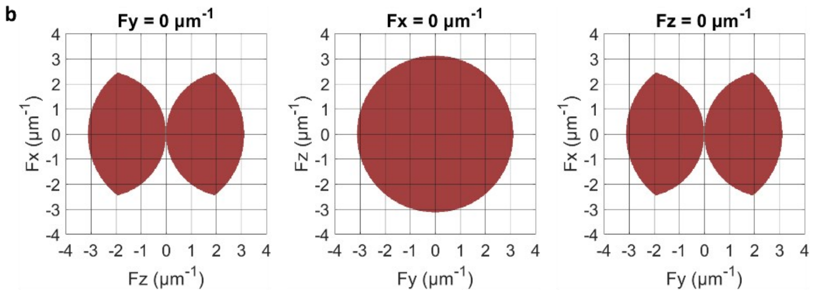

c

	Static Holographic Tomography	Holo-Tomographic Flow Cytometry
$\mathbf{F_y^C}$	4.88 μm^{-1}	3.12 μm^{-1}
$\mathbf{F_z^c}$	1.95 μm^{-1}	3.12 μm^{-1}
$\mathbf{F_x^C}$	2.44 μm^{-1}	2.44 μm^{-1}
**V**	83.37 μm^{-3}	111.50 μm^{-3}

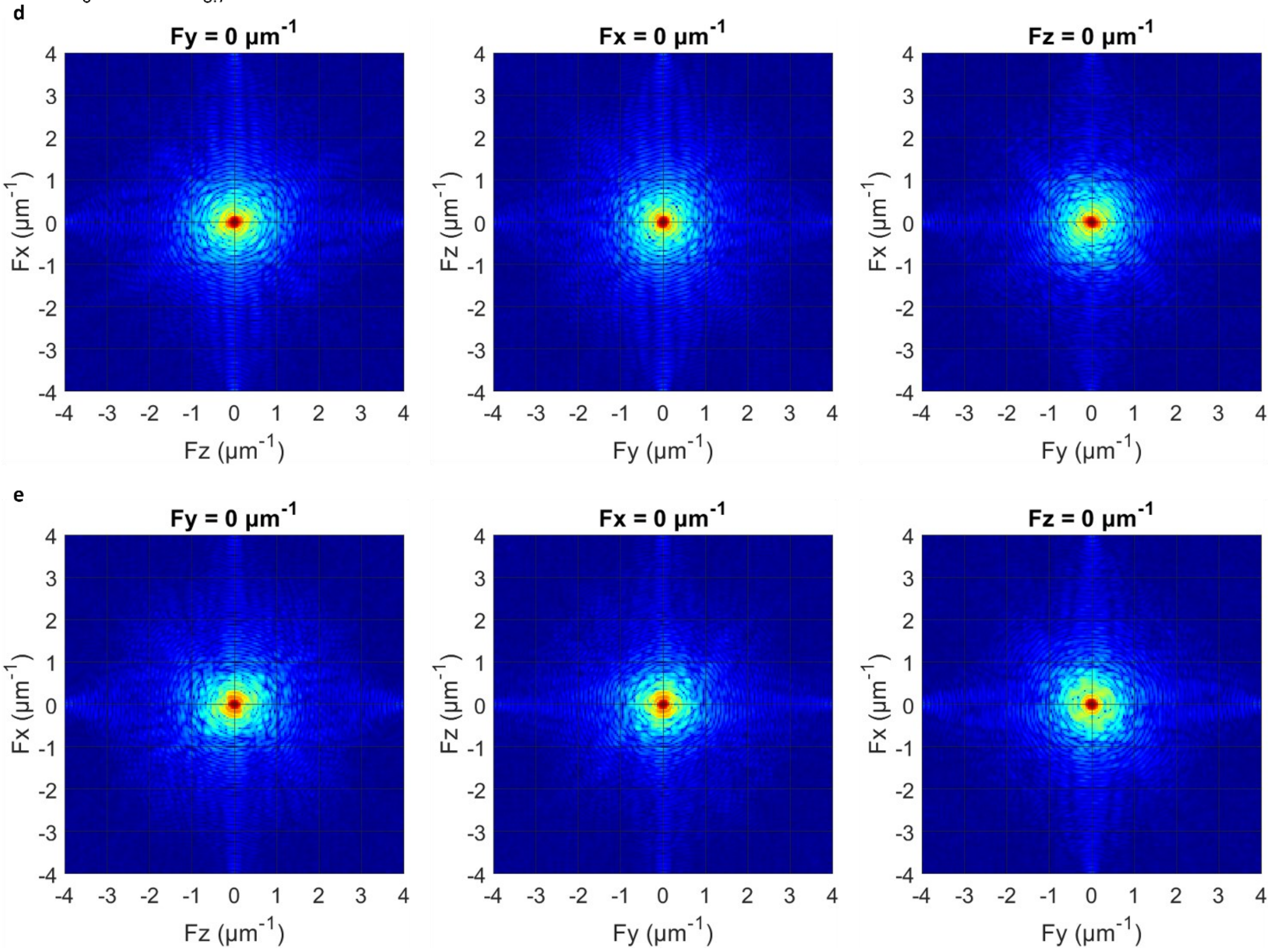

Fig. S4. OTF analysis. (a,b) OTF of the HTFC system and corresponding central slices, respectively. **(c)** Comparison between the OTF of static HT and HTFC in terms of cutoff frequencies (F^C) and OTF volume (V). These values have been computed according to formulas in Ref. [30] by using NA = 1.3 as numerical aperture, n_0 = 1.334 as RI of the surrounding medium, and λ = 0.532 μm as wavelength. For the static HT system based on illumination scanning, the special case of the resolution limit has been considered, i.e. α = 90° and $|\beta| \leq \alpha$. **(d,e)** Central slices of the 3D Fourier transform about the HTFC tomogram of the OCI-AML-3 cell in Fig. S2 and the OCI-AML-2 cell in Fig. S3, respectively.

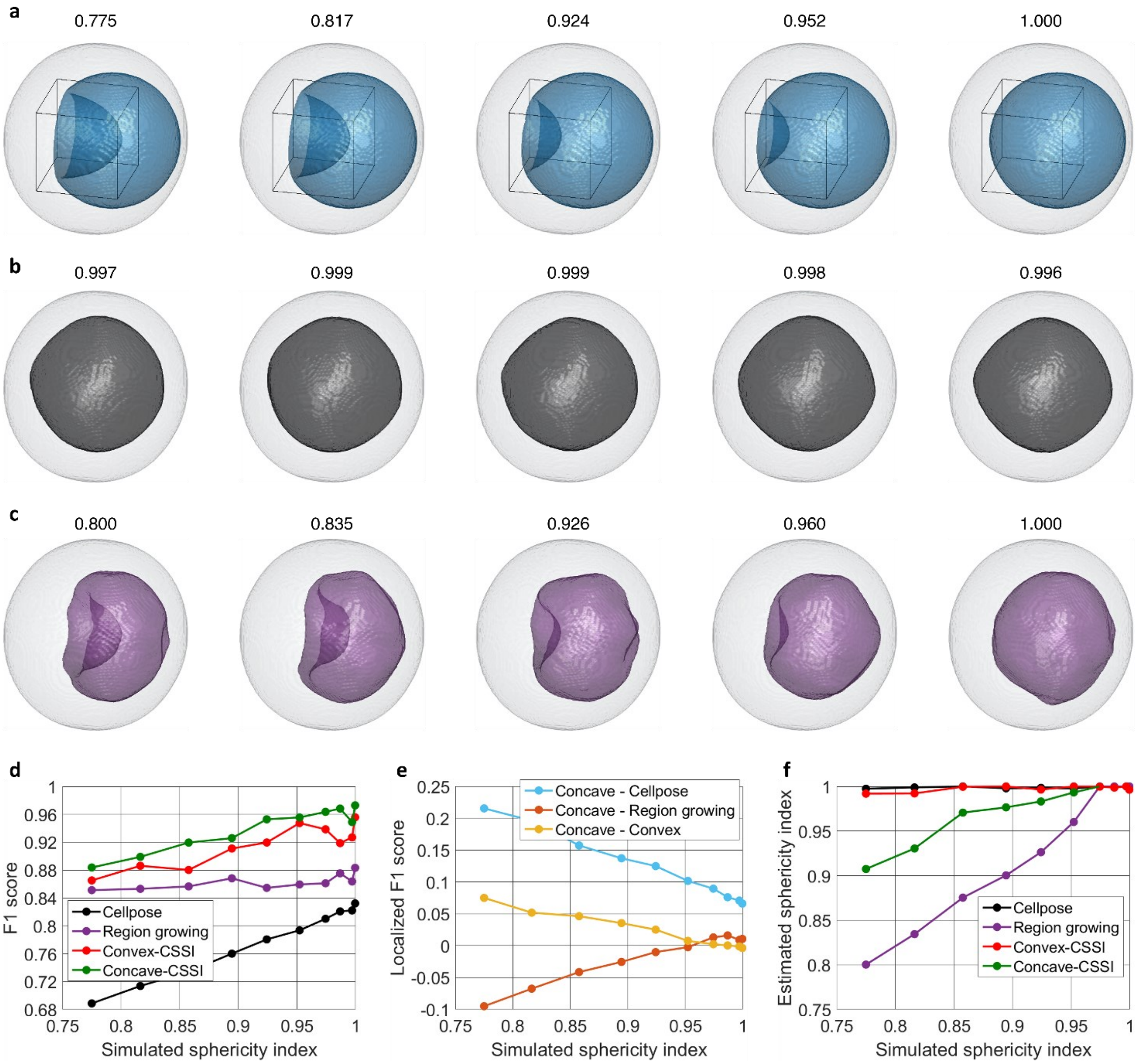

Fig. S5. Comparison between the performance of the CSSI algorithm and two well-established segmentation algorithms based on the 3D numerical cell phantom. **a** Isolevels representation of some simulated nuclei (blue) within the cell shell (gray). **b** Isolevels representation of some nuclei segmented by the Cellpose algorithm (black) within the cell shell (gray). **c** Isolevels representation of some nuclei segmented by the Region Growing algorithm (violet) within the cell shell (gray). In (a-c), the nucleus sphericity indexes are reported at the top. **d** Comparison between the F1 scores about the nucleus segmentations obtained at different simulated sphericity indexes by the Cellpose, Region Growing, convex-CSSI, and concave-CSSI algorithms. **e** Difference between the localized F1 scores about the nucleus segmentations obtained at different sphericity indexes by the concave-CSSI and the Cellpose algorithms, the concave-CSSI and the Region Growing algorithms, and the concave-CSSI and the convex-CSSI algorithms. The VOI is highlighted in (a) by the black box. **f** Comparison between the sphericity indexes estimated from the nucleus segmentations obtained at different simulated sphericity indexes by the Cellpose, Region Growing, convex-CSSI, and concave-CSSI algorithms.

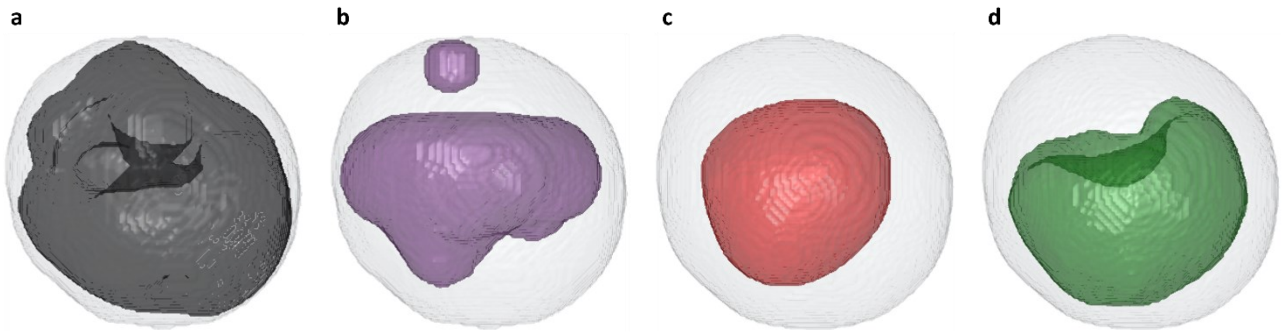

Fig. S6. Experimental validation of the concave-CSSI algorithm with the concave nucleus of an OCI-AML-3 cell reconstructed by HTFC. **a** Cellpose algorithm. **b** Region Growing algorithm. **c** Convex-CSSI algorithm. **d** Concave-CSSI algorithm.

References

S1. Mann, H. B. & Whitney, D. R. On a test of whether one of 2 random variables is stochastically larger than the other. Annals of Mathematical Statistics **18**, 50-60 (1947).

S2. Wilcoxon, F. Individual comparisons by ranking methods. Biometrics Bulletin **1**, 80-83 (1945).

S3. Hÿtch, M. & Hawkes, P. W. Morphological image operators (Academic Press, 2020).
